# Comparing Clinical and Genetic Characteristics of *De Novo* and Inherited *COL1A1/COL1A2* Variants in a Large Chinese Cohort of Osteogenesis Imperfecta

**DOI:** 10.3389/fendo.2022.935905

**Published:** 2022-07-14

**Authors:** Yazhao Mei, Hao Zhang, Zhenlin Zhang

**Affiliations:** Shanghai Clinical Research Center of Bone Disease, Department of Osteoporosis and Bone Diseases, Shanghai Jiao Tong University Affiliated Sixth People’s Hospital, Shanghai, China

**Keywords:** osteogenesis imperfecta, *COL1A1*, *COL1A2*, *de novo*, inherited, clinical scoring system

## Abstract

**Purpose:**

Nearly 85%-90% of osteogenesis imperfecta (OI) cases are caused by autosome dominant mutations of *COL1A1* and *COL1A2* genes, of which *de novo* mutations cover a large proportion, whereas their characteristics remain to be elucidated. This study aims to compare the differences in clinical and genetic characteristics of *de novo* and inherited *COL1A1/COL1A2* mutations of OI, assess the average paternal and maternal age at conception in *de novo* mutations, and research the rate of nonpenetrance in inherited mutations.

**Materials and Methods:**

A retrospective comparison between *de novo* and inherited mutations was performed among 135 OI probands with *COL1A1/COL1A2* mutations. Mutational analyses of all probands and their family members were completed by Sanger sequencing. A new clinical scoring system was developed to assess the clinical severity of OI quantitatively.

**Results:**

A total of 51 probands (37.78%) with *de novo* mutations and 84 probands (62.22%) with inherited mutations were grouped by the results of the parental gene verification. The proportion of clinical type III (*P*<0.001) and clinical scores (*P*<0.001) were significantly higher in *de novo* mutations. Missense mutations covered a slightly higher proportion of *de novo COL1A1* mutations (46.34%) compared with inherited *COL1A1* mutations (33.33%), however, lacking a significant difference (*P*=0.1923). The mean BMD Z/T-score at the lumbar spine in *de novo* mutations was -2.3 ± 1.5, lower than inherited mutations (-1.7 ± 1.8), but lacking statistical significance (*P*=0.0742). There was no significant difference between the two groups in OI-related phenotypes (like fracture frequency, blue sclera, and hearing loss) and biochemical indexes. In *de novo* mutations, the average paternal and maternal age at conception was 29.2 (*P*<0.05) and 26.8 (*P*<0.0001), respectively, which were significantly younger than the average gestational age of the population. Additionally, 98.04% of pedigrees (50/51) with *de novo* mutations were spontaneous conception. The rate of nonpenetrance of parents with pathogenic variants in the inherited mutation group was 25.64% (20/78).

**Conclusions:**

Our data revealed that the proportion of clinical type III and clinical scores were significantly higher in *de novo* mutations than in inherited mutations, demonstrating that *de novo* mutations are more damaging because they have not undergone purifying selection.

## Introduction

Osteogenesis imperfecta (OI) is a generalized connective tissue disorder, apart from liability to slight trauma fractures, the phenotypes usually extend to non-skeletal symptoms, like blue sclera, dentinogenesis imperfecta, hearing loss, and ligamentous laxity (1). Based on the clinical and radiographic manifestations, Sillence et al. (2) in 1979 proposed the classic classification of OI: type I-IV: type I is the mildest kind characterized by nondeforming with persistently blue sclerae; type II is the perinatal lethal form; type III is the severest among surviving patients and presents with progressively deforming; type IV is characterized by white sclerae, whose severity is between type I and type III.

Although an increasing number of genes have been identified in OI involving different modes of inheritance (3), about 85%-90% of OI cases are caused by autosome dominant mutations of *COL1A1* and *COL1A2* genes. They encode the α1(I) and α2(I) chains of type I collagen, which is the main component of the extracellular matrix of bone and skin and is responsible for their elastic properties (4). Mutations in *COL1A1* and *COL1A2* of collagen-related OI can be divided into two groups: quantitative defect (haploinsufficiency) with synthesizing about half the amount of structurally normal type I collagen, primarily resulting from nonsense, frameshift, and some splice-site variants; another is the structural defect, in which over 80% mutations are the results of glycine substitutions in the helical domain (4). The majority of the type II-IV are the consequences of a structural defect because it has more severe impacts on the extracellular matrix than haploinsufficiency does.

Parental genetic characteristics will be passed on to the next generation. At the same time, *de novo* mutations, a kind of novel genetic change in a family, will occur during the formation of the gametes or postzygotic in each of us (5, 6). Only mutations present in the germ cells can be passed on to the next generation (7). Distinct from inherited variants, *de novo* mutations haven’t undergone purifying selection during the reproductive phase, thus making them more harmful (8). Approximately 80% of all *de novo* germline point mutations occur on the paternal allele because oocytes experience only one additional round of DNA replication during their evolution into a mature ovum, whereas spermatogonia will encounter hundreds of DNA replication and cell fission before becoming sperm cells. What’s more, it has been demonstrated that most types of *de novo* mutations are much more correlated with increased paternal age at birth (9–11). Rare sporadic diseases in the population are associated with *de novo* mutations (12). In OI, nearly half of the cases are derived from *de novo* mutations (13–15).

Previous studies have elucidated the genotype-phenotype correlation and mutation spectrum of collagen-related OI through large cohorts (15–18), which contributes to achieving a more accurate and economical diagnosis of OI. However, in addition to some reports of *de novo* mutation cases in OI (16, 19), few large cohort studies have compared *de novo* with inherited mutations in collagen-related OI (14). In this study, we enrolled 135 Chinese probands of OI with *de novo* or inherited *COL1A1/COL1A2* mutations in the department of Osteoporosis and Bone Disease of Shanghai Jiao Tong University Affiliated Sixth People’s Hospital, and then compared the gene mutation spectrum and phenotypic characteristics to investigate differences between *de novo* and inherited mutations among OI with *COL1A1/COL1A2* mutations.

## Materials and Methods

### Subjects

**Figure 1:**
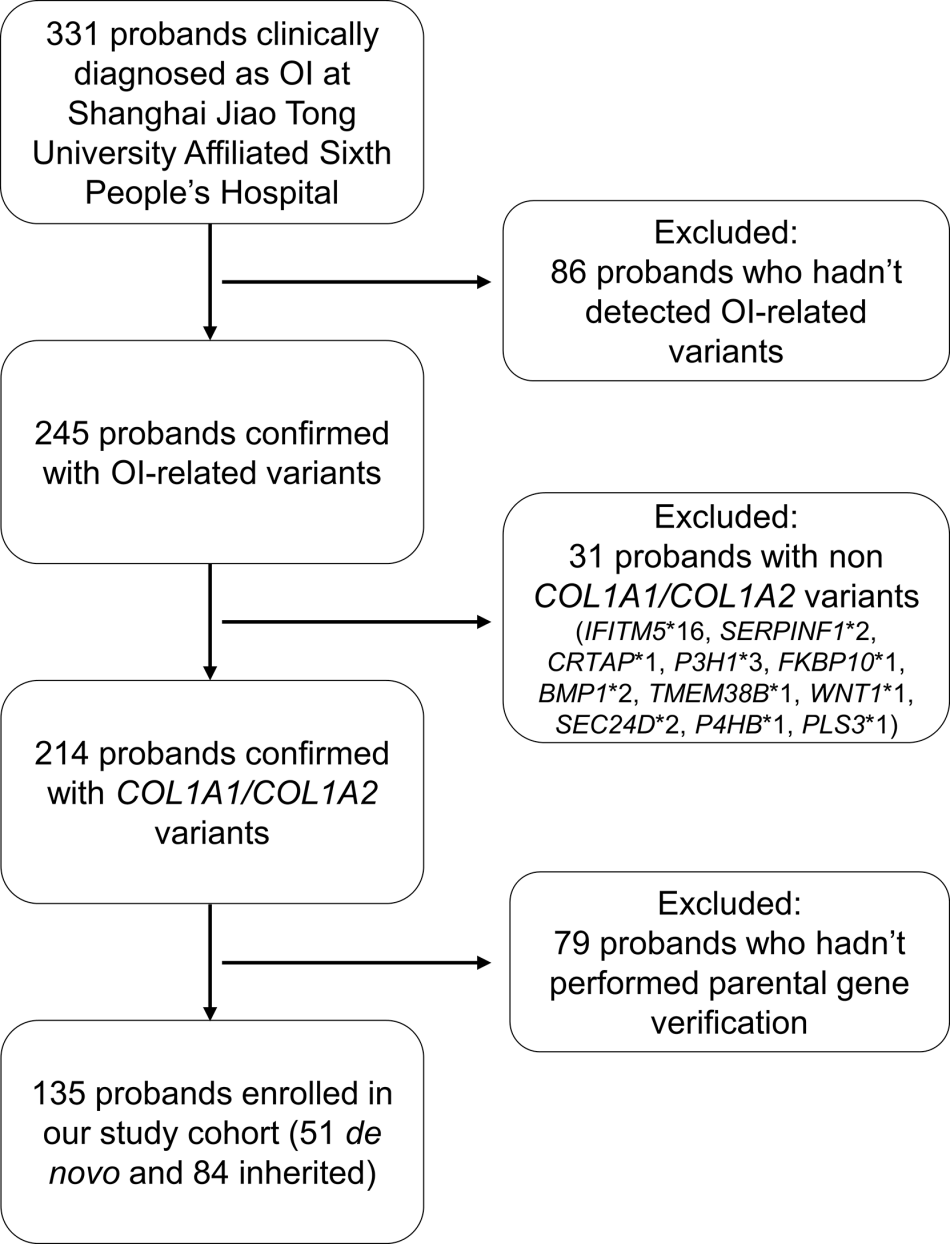
Flowchart of study cohort selection.

We carried out a retrospective review of the electronic medical record from January 2005 to January 2022 for all patients at the department of Osteoporosis and Bone Disease of Shanghai Jiao Tong University Affiliated Sixth People’s Hospital who were clinically diagnosed as OI (**Figure 1**). Our inclusion criteria were (1): probands were confirmed with *COL1A1* or *COL1A2* mutation; (2) pedigrees had undergone parental gene verification. The Ethics Committee of Shanghai Jiao Tong University Affiliated Sixth People’s Hospital approved the study. Written informed consent was obtained from patients or legal guardians of children younger than 18.

### Clinical Evaluation

Clinical information, including age at diagnosis, gender, family history, non-skeletal features (like blue sclera, dentinogenesis imperfecta, and hearing loss), and medical history were collected. As for *de novo* pedigrees, the parental age at conception, methods of conception, and reproductive times were also collected. Information on bone fractures was evaluated, including site, time of initiation, and frequency (per year). The patient’s height was adjusted to standard deviation scores (SDS) based on reference data from the Chinese National Centers for Disease Control and Prevention (20). Baseline bone mineral density (BMD) at the lumbar spine (before bisphosphonate or denosumab treatment) was measured by dual-energy X-ray absorptiometry (DXA) (Lunar, Madison, WI, USA; Hologic, Boston, MA, USA). The coefficient of variability (CV) values of the BMD for the Lunar device at L1-L4 and femoral neck were 1.39% and 2.22%, respectively (21). The CV values of the BMD for the Hologic device at L1-L4 and femoral neck were 0.9% and 0.8%, respectively (21). The results were transformed into age- and sex-specific Z scores (T-scores for males over 50 years old and postmenopausal females) by combing reference data (22–24). In addition, X-ray radiography of the upper and lower extremities and thoracolumbar vertebrae were examined. Then based on the clinical and radiological characteristics, probands were categorized as clinical type I, III, or IV according to the Sillence classification (2). Type II OI was not included in this study as it’s a lethal perinatal form.

Apart from the Sillence classification, we also proposed a new clinical scoring system to assess the severity of OI quantitatively by referring to the scoring criteria of the previous OI researches (18, 25, 26). Clinical score = number of fractures (1-3) + fracture frequency (per year) (1-3) + bowing of lower limbs (0-4) + scoliosis (0-2) + height standard deviation score (SDS) (1-4) + BMD Z/T-score (1-4). The specific contents are displayed in **Table 1**. The higher the clinical score, the more severe the clinical phenotype.

**Table 1:** The proposed clinical scoring system for osteogenesis imperfecta patients.

		Score		Score		Score		Score
Number of fractures	Number<10	1	10 ≤ Number < 30	2	Number ≥ 30	3		
Fracture frequency (per year)	Frequency ≤ 1	1	1 < Frequency < 3	2	Frequency ≥ 3	3		
Bowing of lower limbs	No	0	Mild^a^	2	Severe	4		
Scoliosis	No	0	Mild^a^	1	Severe	2		
Height SDS	SDS ≥ -2	1	-3 ≤ SDS < -2	2	-4 ≤ SDS < -3	3	SDS < -4	4
BMD Z/T-score	Z/T ≥ -2	1	-3 ≤ Z/T < -2	2	-4 ≤ Z/T < -3	3	Z/T < -4	4

a only radiographic findings and no obvious impact on life.

Total score = 20.

Serum levels of calcium (Ca), phosphate (P), and alkaline phosphatase (ALP) were measured by the Hitachi 7600-020 automatic biochemistry analyzer. Bone formation markers osteocalcin (OC), the bone resorption marker serum beta cross-linked C-terminal telopeptide of type 1 collagen (β-CTX), intact parathyroid hormone (PTH), and 25-hydroxyvitamin D (25OHD) were measured using the Cobas 6000 auto-analyzer (Roche Diagnostics, Mannheim, Switzerland).

### *COL1A1/COL1A2* Gene Mutation Analysis *via* Sanger Sequencing

Genomic DNA was extracted from the peripheral blood of all probands and their parents by standard techniques using a DNA extraction kit (Lifefeng Biotech, Shanghai). Exons and exon-intron boundaries of *COL1A1* and *COL1A2* genes were amplified by polymerase chain reaction (PCR) (GenBank accession NO. NC_000017.11 and NO. NC_000007.14). Primers were designed by the Web-based Primer 3 software (https://bioinfo.ut.ee/primer3-0.4.0/). Direct sequencing was performed using BigDye Terminator Cycle Sequencing Ready Reaction Kit, v. 3.1 (Applied Biosystems, California, USA). The products were analyzed with an ABI 3730 sequencer (Foster, CA, United States). SNPs were identified using Polyphred (https://droog.gs.washington.edu/polyphred/). Then, the parental gene verification was performed in the probands’ parents and siblings by Sanger sequencing. Results absent from the Osteogenesis Imperfecta Variant Database (https://oi.gene.le.ac.uk/) were regarded as novel mutations.

### Statistical Analysis

Measurement data (including age at diagnosis, height SDS, BMD Z/T-score, fracture frequency, and clinical score) were presented as mean ± standard deviation, and abnormal distributions were presented as medians (Q1, Q3). The differences between *de novo* and inherited groups were analyzed as appropriate by Grouped T-Test or Non-parametric Test. Enumeration data (like gender and *COL1A1/COL1A2*) were expressed as numbers (%) or ratios, and intergroup differences were evaluated with Chi-Square Test or Continuity Adjustment Chi-Square Test as appropriate. The statistical analyses were performed by SAS 8.2 (SAS Institute Inc., Cary, NC, USA). A two-sided P < 0.05 was considered statistically significant.

## Results

Altogether, 135 probands from 135 unrelated and non-consanguineous pedigrees, including 118 patients who had been published in our previous studies (15, 27, 28), were enrolled in the study, among which 51 probands (37.78%) belonged to the *de novo* mutation group, and the remaining 84 probands (62.22%) were from the inherited mutation group. The details of clinical characteristics of each proband, *COL1A1* and *COL1A2* mutation loci identified in the present study and the parental gene verification are listed in **Supplementary Tables 1**–**4**.

### Clinical Characteristics of Probands in *De novo* and Inherited Mutations

**Figure 2:**
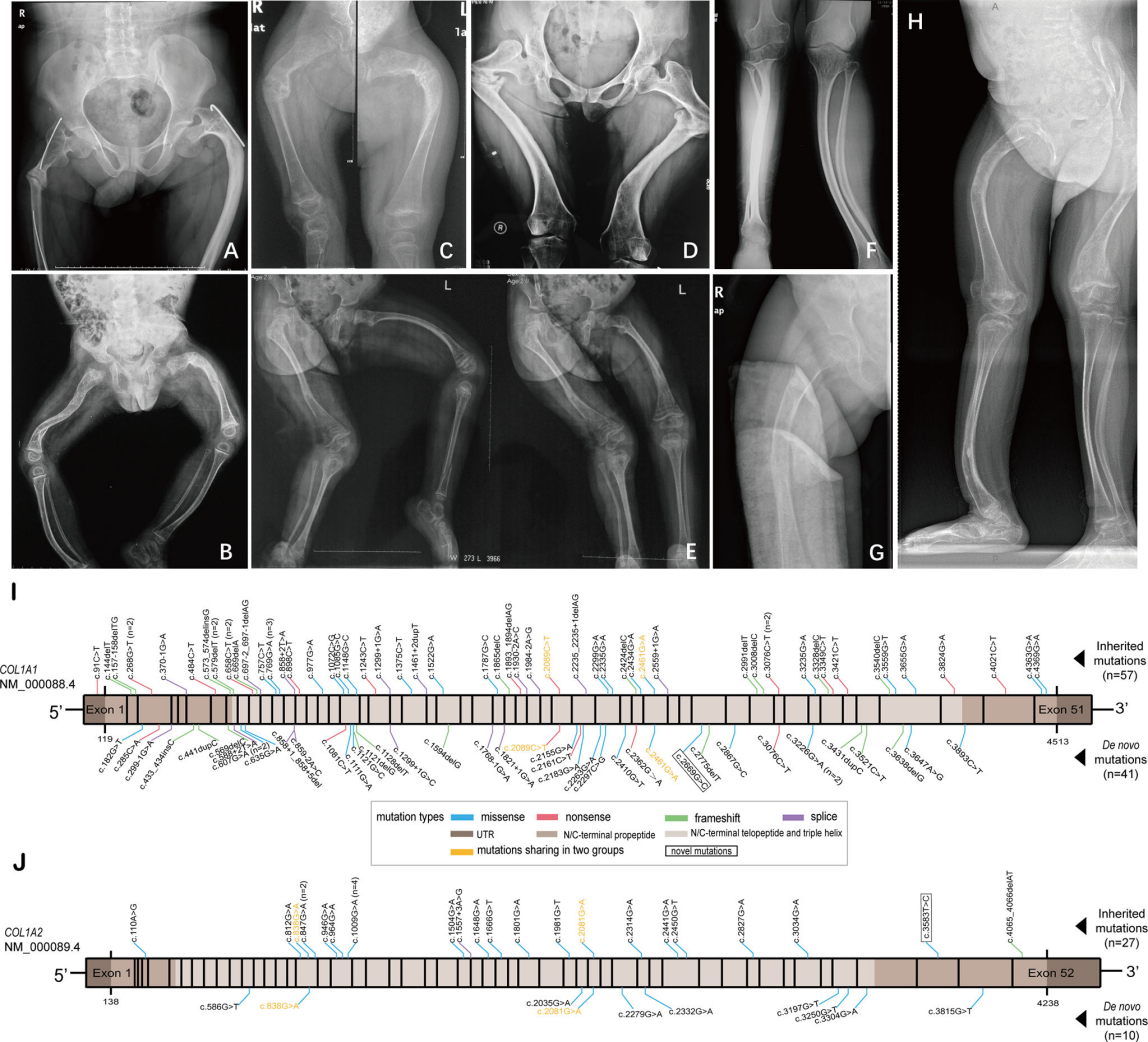
X-ray radiography of the lower extremities of six *de novo* and two inherited mutation probands with clinical type III, and diagrams showing mutation loci identified in this study. **(A, B)** X-ray radiography of the femurs of the probands (p68 and p56) with inherited mutations. **(C–H)** X-ray radiography of the lower extremities of the probands (P9, P3, P49, P43, P13, and P26, respectively) with *de novo* mutations. **(I)** Mutation loci in *COL1A1* gene identified in two groups. **(J)** Mutation loci in *COL1A2* gene identified in two groups.

The comparisons of clinical characteristics of probands between *de novo* and inherited mutations were summarized in **Table 2**. Among 51 *de novo* mutation cases, twenty-one (41.18%) probands with *de novo* mutation had at least one unaffected sibling, and one proband (P37, 1.96%) had an affected twin sister. The mean height SDS in *de novo* mutations was -2.3 ± 3.0, lower than inherited mutations (-1.4 ± 1.9), and the mean BMD Z/T-score at the lumbar spine was -2.3 ± 1.5, also lower than inherited mutations (-1.7 ± 1.8), however, both of them lacking significance (P=0.0767 and 0.0742, respectively). Like inherited mutations, blue sclera covered a substantial proportion of *de novo* mutations (82.22%), while hearing loss was relatively infrequent (2.22%). There were no significant differences in age at diagnosis, gender, three phenotypes (including the blue sclera, dentinogenesis imperfecta, and hearing loss), and fracture frequency between *de novo* and inherited mutations. As for biochemical indexes (Ca, P, ALP, β-CTX, OC, PTH, and 25OHD), no statistical difference was identified, either. X-rays radiography of the lower extremities of six *de novo* and two inherited mutation probands with clinical type III were exhibited in **Figure 2**. Severe lower limb deformities of these probands could be seen, which resulted in limited movement in their daily life. Although there was no significant difference in the proportion of type I and type IV between *de novo* and inherited mutations, the proportion of type III in the *de novo* mutation group was significantly higher than that in the inherited mutation group (*P* =0.0002).

**Table 2:** Clinical and genetic characteristics of *de novo* and inherited *COL1A1/COL1A2* mutations of OI.

			*De novo* (n=51)		Inherited (n=84)			P-value
			Number of available data	Mean ± SD/n (%)/ratio/median(Q1, Q3)	Number of available data	Mean ± SD/n(%)/ratio/median(Q1, Q3)		
Clinical characteristics								
Age at diagnosis (years)			51	13.6 ± 10.3	84	14.5 ± 10.1	0.6221	
Gender (male/female)			51	32/19	84	57/27	0.5434^†^	
Height SDS			41	-2.3 ± 3.0	76	-1.4 ± 1.9	0.0767	
BMD (g/cm^2^)			29	0.6 ± 0.2	64	0.7 ± 0.2	0.5173	
BMD Z/T-score at the lumbar spine			33	-2.3 ± 1.5	64	-1.7 ± 1.8	0.0742	
Fracture frequency (per year)			46	1.6 ± 1.0	76	1.4 ± 0.7	0.1212	
Blue sclera			45	37 (82.22%)	77	60 (77.92%)	0.5702^†^	
Dentinogenesis imperfecta			45	16 (35.56%)	77	24 (31.17%)	0.6185^†^	
Hearing loss			45	3 (2.22%)	77	6 (7.79%)	0.8970^‡^	
Ca (mmol/L)			19	2.5 ± 0.1	34	2.4 ± 0.1	0.1696	
P (mmol/L)			19	1.4 ± 0.3	35	1.4 ± 0.3	0.9899	
ALP (U/L)			19	200.0 (85.0, 289.0)	35	213.0 (103.0, 277.0)	0.8753^¶^	
β-CTX (ng/L)			14	724.0 (347.5, 1286.0)	28	786.2 (428.1, 1154.0)	0.9685^¶^	
OC (ng/mL)			13	57.2 (26.8, 180.1)	27	61.6 (40.2, 95.9)	0.7539^¶^	
PTH (pg/mL)			19	31.3 (18.8, 45.2)	33	37.9 (27.0, 54.4)	0.0569^¶^	
25OHD (ng/mL)			16	19.7 (12.0, 40.0)	32	22.0 (17.1, 30.3)	0.4038^¶^	
Clinical type I			46	26(56.52%)	77	55 (71.43%)	0.0916^†^	
Clinical type III			46	16 (34.78%)	77	6 (7.79%)	0.0002^†^	
Clinical type IV			46	4 (8.70%)	77	16 (20.78%)	0.0789^†^	
Genetic characteristics								
*COL1A1*/*COL1A2*			51	41/10	84	57/27	0.1134^†^	
Missense mutation of *COL1A1*	Total		41	19 (46.34%)	57	19 (33.33%)	0.1923^†^	
	Gly substitution		19	14 (73.68%)	19	14 (73.68%)	>0.9999^†^	
Missense mutation of *COL1A2*	Total		10	10 (100.00%)	27	25 (92.59%)	>0.9999^†^	
	Gly substitution		10	9 (90.00%)	25	23 (92.00%)	>0.9999^†^	
Clinical score	Total		32	9.1 ± 4.7	63	5.8 ± 2.0	0.0005	
	*COL1A1*	Total	26	8.2 ± 4.6	44	5.6 ± 1.8	0.0092	
		Structural mutation	11	12.6 ± 4.0	13	5.8 ± 1.9	0.0002	
	*COL1A2*	Total	6	13.0 ± 3.2	19	6.2 ± 2.3	0.0020	

Data are shown as mean ± standard deviation, numbers (%), ratios, or median (Q1, Q3). ^†^Chi-Square Test, ^‡^Continuity Adjustment Chi-Square Test, ^¶^Non-parametric Test, otherwise Grouped T-Test.

Bold P values indicate significance.

SDS, standard deviation scores; BMC, bone mineral content; BMD, bone mineral density; Ca, calcium; P, phosphate; ALP, alkaline phosphatase; β-CTX, beta cross-linked C-terminal telopeptide of type 1 collagen; OC, osteocalcin; PTH, intact parathyroid hormone; 25OHD, 25-hydroxyvitamin D.

A total of 20 probands’ fathers or mothers confirmed with pathogenic variants among 84 hereditary cases exhibited no OI-related phenotype or fracture history, that is, nonpenetrance (listed in **Supplementary Table 3**). The available data of parents’ medical history was 78. Hence, the rate of nonpentrance in the inherited mutation group was 25.64% (20/78).

### Genetic Analysis of *COL1A1*/*COL1A2* in *De Novo* and Inherited Mutations

All pedigrees in our study had completed parental gene verification to confirm the mutation mode. A total of 123 different collagen type I variants were identified in 135 probands, including 65 (52.85%) missense mutations, 20 (16.26%) nonsense mutations, 21 (17.07%) frameshift mutations, and 17 (13.82%) splice mutations (**Figures 2I, J**). Of the 123 mutations, two variants (c.2669G>C in *COL1A1*, and c.3583T>C in *COL1A2*) absent in the Osteogenesis Imperfecta Variant Database (https://www.le.ac.uk/genetics/collagen/index.html) were novel mutations.

The mutation spectrum of this study comprised four kinds of mutation types, including missense, nonsense, frameshift, and splice mutation. The comparisons between *de novo* and inherited mutation spectrum were summed up in **Table 2**. *COL1A1* and *COL1A2* mutation cases covered a similar proportion in the *de novo* and inherited groups (*P*=0.1134). Probands with *COL1A1 de novo* mutations were 41, containing 19 (46.34%) missense mutations, and probands with *COL1A1* inherited mutations were 57, including 19 (33.33%) missense mutations. The missense mutation in the *de novo COL1A1* mutation group occupied a larger percentage than in the inherited *COL1A1* mutation group, but lacking significance (*P*=0.1923). All ten probands with *COL1A2 de novo* mutations were missense mutations, and 92.59% of twenty-seven probands with *COL1A2* inherited mutations were missense mutations. No significant intergroup difference in the proportion of missense mutation of *COL1A2* was discovered (*P*>0.9999). Glycine substitutions covered no significantly different proportion of missense mutations in *de novo* and inherited *COL1A1/COL1A2* mutations. Additionally, p.Gly337Ser was seen four times in the *COL1A2* inherited mutation group. Intriguingly, mutations of p.Gly821Ser, p.Arg697*, and p.Arg1026* in *COL1A1*, and p.Gly694Asp in *COL1A2* shared in *de novo* and inherited mutation groups (**Table 3**). The concrete proportions of each mutation type in *COL1A1/COL1A2* for two groups were displayed in **Supplementary Figure 1**.

**Table 3:** Mutations shared in *de novo* and inherited mutation groups.

Gene	Amino acid change	Mutation effect	*De novo*				Inherited			
			Frequency	Proband ID	Clinical type	Clinical score	Frequency	Proband ID	Clinical type	Clinical score
*COL1A1*	p.Gly821Ser	Missense	1	P5	III	15	1	p5	III	N/A
	p.Arg697*	Nonsense	1	P30	IV	4	1	p20	I	5
	p.Arg1026*	Nonsense	1	P19	I	4	2	p8	N/A	N/A
								p52	I	6
*COL1A2*	p.Gly694Asp	Missense	1	P44	III	15	1	p68	III	12

N/A, not available.

### Genotype-Phenotype Correlations in *De Novo* and Inherited Mutations

**Figure 3:**
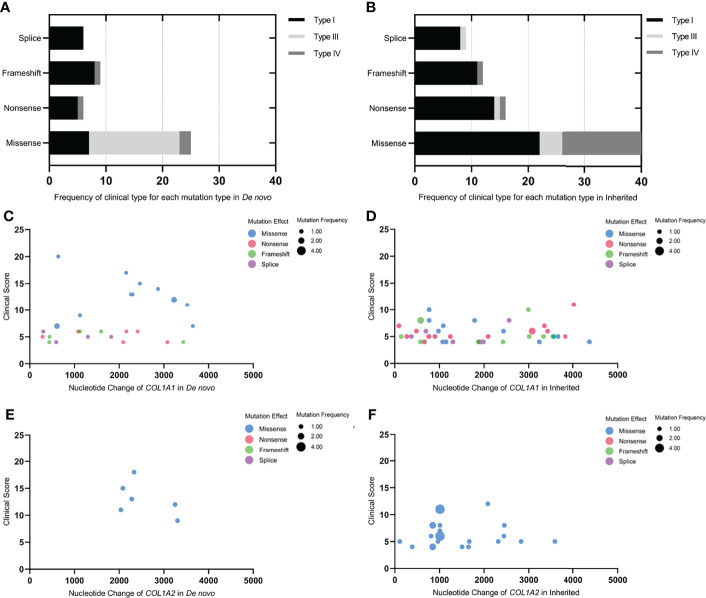
Genotype-phenotype correlations in *de novo* and inherited mutations. **(A, B)** Frequency of clinical type for each mutation type in *De novo* and Inherited. **(C–F)** The bubble plots of the clinical score of available probands with *de novo* or inherited *COL1A1/COL1A2* mutations. X-axis represented the mutation site on the *COL1A1/COL1A2* gene; Y-axis was the clinical score; the colors of the bubble represented different mutation effects (missense, nonsense, frameshift, and splice, respectively); the size of the bubble indicated the mutation frequency of each mutation site in the *de novo* or inherited mutation group. The bubble was higher and bigger; the phenotype was more severe, and the mutation site was more frequent.

The frequency of clinical type in each mutation type in *de novo* and inherited mutations was exhibited in **Figure 3**. Both in *de novo* and inherited mutation groups, mutation types of nonsense, frameshift, and splice mainly resulted in clinical type I, and clinical type III and IV (especially type III) were primarily correlated with missense mutations. Compared with inherited mutations in *COL1A1/COL1A2*, probands with *de novo* mutations had significantly higher clinical scores (*P* = 0.0005) (**Table 2**), which denoted more severe phenotypes. We also compared clinical scores about *COL1A1*(*P* = 0.0092), missense mutation of *COL1A1*(*P*=0.0002), and *COL1A2*(*P*=0.0020) between two groups, respectively, and their significant results were consistent with the outcome mentioned above. At the same time, we presented clinical scores of available probands on different *de novo* or inherited *COL1A1/COL1A2* mutation loci with the help of bubble plots (**Figure 3**). From four different bubble plots, we could find that: firstly, there was no noticeable difference in the mutation site distribution for the *COL1A1/COL1A2* gene between *de novo* and inherited mutations; secondly, the clinical score of missense mutation was higher than the other three mutation effects in general; lastly, bubbles of the *de novo COL1A1/COL1A2* mutations distributed higher in the axis than the inherited did, which means the clinical scores of *de novo* mutations were higher and phenotypes were more severe. Four mutation loci shared in *de novo* and inherited mutation groups, mentioned above, had a consistent clinical type and clinical score between the two groups, except for one case with p.Arg1026* mutation (**Table 3**).

### The Parental Age at Conception in *De Novo* Mutations

In the *de novo* mutation group, the average paternal and maternal age was 29.2 and 26.8, respectively. In the US, National Vital Statistics System data revealed that the average paternal age at birth was 30.9 in 2015 (29), which was older than the mean paternal age at conception in our study (*P* = 0.0236). The average maternal age at conception in China in 2017 was 29.7 (30), which was also older than our research data (*P <*0.0001). Additionally, except for one proband (P36) who was the outcome of medically assisted reproduction, the remaining 50 pedigrees (98.04%) with *de novo* mutations were spontaneous conception.

## Discussion

Previous studies had illustrated the proportion of *de novo* mutations in OI with *COL1A1*/*COL1A2* mutations: 41.03% in South Korea (13), 56.16% in Estonia, Ukraine, and Vietnam (14), and 54.6% in our previous report (15). The percentage of *de novo* mutations in our study (37.78%) was significantly lower than our last report (*P* = 0.0029), which may be due to we excluded cases without performing parental gene verification. OI is a phenotypically heterogeneous disease that familial cases with the same mutation can present remarkably phenotypic diversity, ranging from mild to severe (31, 32). In our study, the rate of nonpenetrance of hereditary probands’ parents was up to 25.64%. Therefore, some affected parents of hereditary probands exhibited very mild or even without symptoms, which may result in missed diagnosis and increasing the false-positive rate of *de novo* mutations if not performing parental gene verification.

Low bone mass is always presented in patients with OI, and in our study, the *de novo* mutation group exhibited a lower BMD Z/T-score at the lumbar spine, however, not significantly lower than the inherited group. Fractures of long bones and vertebral compression are the most notable features of OI, while there was no difference in the fracture frequency between *de novo* and inherited mutation groups in our study. Skeletal deformities like bowing of long bones and scoliosis are usually associated with severe type III. Apart from congenital causes, recurrent fractures of extremities can also result in deformities. As displayed in **Figure 2**, lower limb deformities would seriously damage the ability to move, and most of them had no alternative but to be in a wheelchair for the rest of their lives. Most of our patients’ severe lower limb deformities were accompanied by severe scoliosis. Activity limitations and participation restrictions are linked to lower quality of life (QOL) (33), and physical QOL is worse in children with more severe OI than in mildly affected children. In an aspect of non-skeletal manifestation, blue sclera was the most prevalent feature of patients with OI (28), and consistent with the previous studies (34, 35), dentinogenesis imperfecta covered a higher proportion in type III and IV OI than type I in our study. Biochemical indexes including bone turnover markers showed no statistical differences in our study. Several papers have revealed that there were no significant differences in levels of the bone turnover markers between different OI clinical types or different OI mutation types (36, 37), therefore, the relationship between severity and bone turnover rates in OI may be more complicated.

Generally, haploinsufficiency of *COL1A1* results in the mildest form of OI. In contrast, haploinsufficiency of *COL1A2* results in no apparent OI-related phenotype, and homozygous null mutations of *COL1A2* bring about phenotypes of varying severity (38). However, in our study, one case (p61, c.1557+3A>G) with haploinsufficiency of *COL1A2* mutation showed severe phenotypes and was classified as type III. Most of the structural defects are missense mutations, in which over 80% of mutations are the results of glycine substitutions in the Gly-Xaa-Yaa repeat (4). The most frequent cause of OI with *COL1A2* mutation is the structural defect of type I collagen, especially glycine substitutions in the helical domain (16, 39). Consistently, both *de novo* and inherited *COL1A2* mutations in our study were predominantly composed of missense mutations (100% and 92.59%, respectively), especially glycine substitutions. It has been widely demonstrated that structural mutations have more severe clinical consequences than quantitative mutations, and clinical type II-IV are usually caused by mutations altering structures (1, 4). Similarly, clinical type III was mainly driven by missense mutations and type I primarily resulted from quantitative defects in our study, both in *de novo* and inherited mutation groups. Repeated mutations are often associated with CpG dinucleotides, described as mutational hotspots (40). The mutation of p.Gly337Ser in *COL1A2* was detected in four unrelated families in our study, that is, mutational hot spots. Four different mutation loci mentioned above were shared in *de novo* and inherited mutation groups. Almost all of them had a consistent clinical type and clinical score between the two groups, indicating that the severity of the same mutant locus did not differ between *de novo* and inherited mutations.

Our results revealed that the proportion of type III and clinical scores were significantly higher in the *de novo* mutation group. *De novo* mutations are genetically different from inherited mutations and are more damaging because mutation processes in *de novo* mutations are happening between generations without undergoing purifying selection (41). Zhytnik et al. (14) revealed that in collagen-related OI, missense mutations and type III occupied a larger proportion in *de novo* mutations, and type I was lower compared with inherited mutations. In our study, the missense mutations in the *de novo COL1A1* mutation group covered a higher proportion than in the inherited *COL1A1* mutation group, though lacking significance, which may be a reason why there were more type III cases and higher clinical scores in the *de novo* mutation group. Although there was no difference in the mutation spectrum between the *de novo* and inherited *COL1A2* mutation groups, type III cases and clinical scores were still higher in *de novo* mutations than in inherited *COL1A2* mutations, probably due to mutation loci in *de novo* mutations with more serious pathogenicity since they have not undergone purifying selection. Additionally, these significant results confirmed that our clinical scoring system could quantitatively reflect the clinical severity of OI. Errors in DNA replication, exposure to mutagens, or failure to repair DNA damage can result in *de novo* mutations (12). Nowadays, advanced paternal age at conception has been considered the predominant factor associated with an increasing number of *de novo* mutations (9, 11, 42, 43). However, in our study, the average paternal age at conception was 29.2 years, significantly younger than the data of the US (29). Thus, paternal age may not be a critical factor contributing to *de novo* mutations in our research. In addition, Wong et al. (44) reported that medically assisted conception would increase the incidence of *de novo* mutations compared with natural conception. However, recently, a pilot study by Smits et al. (11) showed no impact of assisted reproductive technologies on the number of *de novo* mutations in the genome of the offspring. In our study, the majority of the pedigrees (98.04%) with *de novo* mutations were spontaneous conception, which limited us to investigate the impact of different methods of conception on *de novo* mutations.

Mosaicism means an individual develops from a single fertilized egg but has two or more genetically distinct cell populations (45). Postzygotic mutations, generated in the first few cell divisions after fertilization, can result in mosaicism and exist in many different tissues (43). It has been estimated that approximately 7% of *de novo* mutations are early postzygotic events with high-level mosaicism in the blood (46–48). The timing of the postzygotic mutations is of vital importance as it determines the proportion of affected tissues and phenotypes (45). The recurrence risk is very low, the same as the population risk, in parents having a child with a postzygotic mutation (45). Some *de novo* mutations, but actually because of the parental germline mosaicism, can be shared by more than one sibling. The higher the proportion of mosaicism in parental blood cells, the higher the recurrence risk among siblings (43). Furthermore, some mutations that arise in the germ cell lineage during early embryonic development are undetectable in somatic cells like blood or buccal mucosa, but can also be passed on to the next generation (7). Halvorsen et al. (49) revealed that germline mosaic mutations, which can be transmitted, have not had to undergo purifying selection as *de novo* mutations. Pyott et al. (50) found that the recurrence of lethal OI usually results from parental mosaicism. Therefore, *de novo* mutations actually resulting from paternal germline mosaicism have meaningful necessities for genetic counseling about recurrence risks. The detection limit of Sanger sequencing is about 15-20% of somatic mosaic variants. Therefore, deep sequencing, like SNaPshot minisequencing assay or whole-exome sequencing, is recommended to determine the percentage of mosaicism.

Although our comparative study has carried out a meticulous comparison between *de novo* and inherited *COL1A1/COL1A2* mutations of OI, there are still some limitations to the study that must be considered: (1) This study was a retrospective review and suffers from the limitations inherent in such a design. (2) By adhering strictly to the inclusion criteria, we removed over 36% of probands in our institution who were confirmed with *COL1A1/COL1A2* pathogenic mutations, which may have resulted in differences in the prevalence of *de novo* mutations we report when compared with other studies, and influence the clinical and genetical differences in two groups. However, our primary purpose was to compare the difference between two mutational patterns in OI, thus we must be 100% confident with the mode of inheritance of each proband in our study cohort. Therefore, we consider this as less of a limitation and more of a strength of the study. (3) We have not adopted deep sequencing in parental samples to make it clear whether some *de novo* mutations were actually caused by parental germinal mosaicism. (4) The research about non-skeletal phenotypes is not comprehensive in our study, for instance, cardiovascular abnormalities.

## Conclusion

Our study showed that the proportion of clinical type III and clinical scores were significantly higher in *de novo* mutations compared with inherited *COL1A1* mutations, which may demonstrate that the pathogenicity of *de novo* mutations is, on the whole, more severe than inherited mutations because *de novo* variants have not undergone purifying selection. These findings highlight the further need to investigate the differences between *de novo* and inherited mutations in OI and to research the occurrence of germline mosaicism in the parents of *de novo* mutations, which can provide accurate genetic counseling for both early prenatal and preimplantation diagnosis.

## Data Availability Statement

The original contributions presented in the study are included in the article/**Supplementary Material**. Further inquiries can be directed to the corresponding authors.

## Ethics Statement

The studies involving human participants were reviewed and approved by The Ethics Committee of Shanghai Jiao Tong University Affiliated Sixth People’s Hospital. Written informed consent to participate in this study was provided by the participants’ legal guardian/next of kin. Written informed consent was obtained from the individual(s), and minor(s)’ legal guardian/next of kin, for the publication of any potentially identifiable images or data included in this article.

## Author Contributions

ZZ, and HZ designed the research, and revised the manuscript. YM summarized the clinical data, analyzed the sequencing data, and drafted the manuscript. HZ contributed to funding acquisition. All authors contributed to the article and approved the submitted version.

## Funding

This research was funded by the National Natural Science Foundation of China (81870618).

## Conflict of Interest

The authors declare that the research was conducted in the absence of any commercial or financial relationships that could be construed as a potential conflict of interest.

## Publisher’s Note

All claims expressed in this article are solely those of the authors and do not necessarily represent those of their affiliated organizations, or those of the publisher, the editors and the reviewers. Any product that may be evaluated in this article, or claim that may be made by its manufacturer, is not guaranteed or endorsed by the publisher.

## Supplementary Material

Supplementary Figure 1
*De novo* and inherited mutation spectrum of *COL1A1*/*COL1A2*. **(A)** *De novo* mutation spectrum of *COL1A1.* **(B)** *De novo* mutation spectrum of *COL1A2.* **(C)** Inherited mutation spectrum of *COL1A1.* **(D)** Inherited mutation spectrum of *COL1A2.*

Supplementary Table 1 Clinical characteristics of *De novo* mutations.

Supplementary Table 2 Mutations in *COL1A1/COL1A2* of probands with *de novo* mutations.

Supplementary Table 3 Clinical characteristics of *De novo* mutations.

Supplementary Table 4 Mutations in *COL1A1/COL1A2* of probands with inherited mutations.
