# Supplementary material for: Comparing Clinical and Genetic Characteristics of De Novo and Inherited COL1A1/COL1A2 Variants in a Large Chinese Cohort of Osteogenesis Imperfecta
Source: Front Endocrinol (Lausanne). 2022 Jul 14;13:935905. doi: 10.3389/fendo.2022.935905 (PMC9329653; doi:10.3389/fendo.2022.935905)
Supplement: Supplementary Figure 1 — De novo and inherited mutation spectrum of COL1A1/COL1A2. (A) De novo mutation spectrum of COL1A1. (B) De novo mutation spectrum of COL1A2. (C) Inherited mutation spectrum of COL1A1. (D) Inherited mutation spectrum of COL1A2. [file DataSheet_1.zip › Supplementary material/Supplementary Table 1.docx]

**SUPPLEMENTARY TABLE 1**┃Clinical characteristics of *De novo* mutations

| **Proband ID** | **Age at diagnosis** | **Sex** | **Consanguinity** | **Parents’ pregnant age** | | **Method of conception** | **Siblings** | **Height(cm)** | **SDS** | **BMD** | | **Fractures** | | **Bowing of lower limbs** | | **Scoliosis** | | **BS** | **DI** | **HL** | **Ca** | **P** | **ALP** | **β-CTX** | **OC** | **PTH** | **25OHD** | **Clinical type** | **Clinical score** |
| --- | --- | --- | --- | --- | --- | --- | --- | --- | --- | --- | --- | --- | --- | --- | --- | --- | --- | --- | --- | --- | --- | --- | --- | --- | --- | --- | --- | --- | --- |
|  |  |  |  | **Father** | **Mother** |  |  |  |  | **LS-BMD (g/cm2)** | **Z/T-score** | **Total** | **Frequency (per year)** | **Mild** | **Severe** | **Mild** | **Severe** |  |  |  |  |  |  |  |  |  |  |  |  |
| P1 | 23.4 | F | No | 22 | 22 | S | one unaffected brother | 156 | -0.9 | 0.841 | -2.1 | 12 | 1 | - | - | - | - | + | + | - | N.A. | N.A. | N.A. | N.A. | N.A. | N.A. | N.A. | I | 6 |
| P2 | 13 | M | No | 28 | 29 | S | No | 116 | -5.6 | 0.242 | -4.9 | 30+ | 4 | - | + | - | + | + | - | - | 2.52 | 1.31 | 394 | 1060 | N.A. | 22.89 | 37.38 | III | 20 |
| P3 | 28 | F | No | N.A. | N.A. | S | one unaffected sister | 140 | -3.8 | N.A. | -2.0 | 11 | 1 | - | + | - | - | + | - | - | 2.6 | 1.6 | 278 | 636 | 84.23 | 23.37 | 19.87 | III | 11 |
| P4 | 35 | M | No | 27 | 25 | S | Two unaffected brothers | 158 | -2.4 | 0.715 | -3.0 | 6 | 2 | - | - | - | - | + | - | + | N.A. | N.A. | N.A. | N.A. | N.A. | N.A. | N.A. | I | 7 |
| P5 | 41 | M | No | 34 | 30 | S | one unaffected sister | 124 | -8.0 | 0.692 | -3.1 | 20+ | 1 | - | + | - | + | + | + | - | N.A. | N.A. | N.A. | N.A. | N.A. | N.A. | N.A. | III | 15 |
| P6 | 25 | M | No | N.A. | N.A. | S | Three unaffected siblings | 100 | -11.9 | N.A. | N.A. | 20+ | 2 | - | - | - | - | + | + | - | N.A. | N.A. | N.A. | N.A. | N.A. | N.A. | N.A. | I | N.A. |
| P7 | 29.1 | M | No | 28 | 31 | S | No | 178 | 0.9 | 0.775 | -2.8 | 10 | 1 | - | - | - | - | + | + | - | N.A. | N.A. | N.A. | N.A. | N.A. | N.A. | N.A. | I | 6 |
| P8 | 4 | F | No | N.A. | N.A. | S | No | N.A. | N.A. | N.A. | N.A. | N.A. | N.A. | N.A. | N.A. | N.A. | - | N.A. | N.A. | N.A. | 2.35 | 0.77 | 97 | N.A. | N.A. | 31.33 | N.A. | N.A. | N.A. |
| P9 | 4 | F | No | 30 | 29 | S | No | N.A. | N.A. | N.A. | N.A. | N.A. | N.A. | - | + | - | - | N.A. | N.A. | N.A. | N.A. | N.A. | N.A. | N.A. | N.A. | N.A. | N.A. | III | N.A. |
| P10 | 11.5 | M | No | 21 | 25 | S | one unaffected brother | 112 | -5.2 | 0.330 | -4.4 | 20 | 2 | - | + | + | - | + | + | - | N.A. | N.A. | N.A. | N.A. | N.A. | N.A. | N.A. | III | 17 |
| P11 | 4 | M | No | 29 | 27 | S | No | N.A. | N.A. | N.A. | N.A. | 5 | 2 | - | - | - | - | + | - | - | N.A. | N.A. | N.A. | N.A. | N.A. | N.A. | N.A. | I | N.A. |
| P12 | 13.8 | M | No | 28 | 26 | S | No | 151.1 | -1.9 | 0.647 | -1.0 | 7 | 1 | - | - | - | - | + | - | - | N.A. | N.A. | N.A. | N.A. | N.A. | N.A. | N.A. | I | 4 |
| P13 | 6 | F | No | 38 | 29 | S | Two unaffected siblings | 120 | 0.7 | N.A. | -2.0 | 6 | 2 | - | + | - | - | + | - | - | N.A. | N.A. | N.A. | N.A. | N.A. | N.A. | N.A. | III | 9 |
| P14 | 25.4 | F | No | 27 | 24 | S | one unaffected sister | 126 | -6.4 | 1.254 | 1.8 | 10+ | 1 | - | + | - | + | + | + | - | N.A. | N.A. | N.A. | N.A. | N.A. | N.A. | N.A. | III | 14 |
| P15 | 11 | M | No | 28 | 27 | S | No | 120 | -3.8 | N.A. | N.A. | 20+ | 2 | + | - | + | - | + | + | - | 2.39 | 1.64 | 270 | N.A. | N.A. | 18.77 | N.A. | I | N.A. |
| P16 | 13.3 | F | No | 23 | 23 | S | one unaffected brother | 150 | -1.6 | 0.602 | -2.3 | 10+ | 1 | - | - | - | - | + | - | - | N.A. | N.A. | N.A. | N.A. | N.A. | N.A. | N.A. | I | 6 |
| P17 | 2 | M | No | 35 | 28 | S | one unaffected sister | 81 | -2.2 | N.A. | N.A. | 4 | 2 | - | - | - | - | + | - | - | N.A. | N.A. | N.A. | N.A. | N.A. | N.A. | N.A. | I | N.A. |
| P18 | 26.7 | F | No | 30 | 31 | S | one unaffected sister | 134.5 | -4.8 | 0.636 | -3.4 | 5 | 1 | - | + | - | - | N.A. | N.A. | N.A. | N.A. | N.A. | N.A. | N.A. | N.A. | N.A. | N.A. | III | 13 |
| P19 | 14 | M | No | 23 | 22 | S | one unaffected sister | 159.5 | -0.9 | 0.723 | -0.4 | 7 | 1 | - | - | - | - | + | - | - | N.A. | N.A. | N.A. | N.A. | N.A. | N.A. | N.A. | I | 4 |
| P20 | 6 | M | No | 37 | 31 | S | No | 117 | -0.2 | N.A. | N.A. | 5 | 1 | + | - | - | - | + | - | - | N.A. | N.A. | N.A. | N.A. | N.A. | N.A. | N.A. | I | N.A. |
| P21 | 2.6 | M | No | 35 | 35 | S | one unaffected brother | 88 | -1.6 | N.A. | N.A. | 5 | 2 | - | - | - | - | + | - | - | N.A. | N.A. | N.A. | N.A. | N.A. | N.A. | N.A. | I | N.A. |
| P22 | 7.6 | M | No | 30 | 27 | S | No | 120 | -1.5 | 0.362 | -3.3 | 2 | 1 | - | - | - | - | + | - | - | N.A. | N.A. | N.A. | N.A. | N.A. | N.A. | N.A. | I | 6 |
| P23 | 2.5 | M | No | 32 | 30 | S | No | 101 | 2.1 | N.A. | N.A. | 2 | 2 | - | - | - | - | + | - | - | N.A. | N.A. | N.A. | N.A. | N.A. | N.A. | N.A. | I | N.A. |
| P24 | 12 | M | No | 32 | 23 | S | No | 150 | -0.3 | N.A. | N.A. | 6 | 2 | - | - | - | - | + | - | - | 2.57 | 1.58 | 198.8 | 1230 | 57.18 | 11.62 | 8.97 | I | N.A. |
| P25 | 12.1 | M | No | 26 | 22 | S | No | 150.8 | -0.2 | 0.459 | -2.6 | 6 | 1 | - | - | - | - | + | - | - | N.A. | N.A. | N.A. | N.A. | N.A. | N.A. | N.A. | I | 5 |
| P26 | 8 | F | No | 40 | 37 | S | one unaffected brother | 104.4 | -4.5 | N.A. | -2.0 | 10+ | 2 | - | + | - | - | + | - | - | N.A. | N.A. | N.A. | N.A. | N.A. | N.A. | N.A. | III | 13 |
| P27 | 3 | F | No | 27 | 24 | S | one unaffected sister | 110 | 3.8 | 0.633 | 0.8 | 10+ | 5 | - | - | - | - | + | + | - | 2.78 | 1.65 | 262 | N.A. | N.A. | 19.3 | N.A. | I | 7 |
| P28 | 13 | M | No | 26 | 24 | S | one unaffected brother | 164 | 0.6 | 0.605 | -1.8 | 10 | 2 | - | - | - | - | - | - | - | 2.3 | 1.36 | 200 | 1455 | 288.4 | 53.23 | 11.7 | IV | 6 |
| P29 | 4 | M | No | 27 | 29 | S | one unaffected brother | 106.1 | 0.5 | 0.504 | -2.0 | 6 | 2 | - | - | - | - | + | - | - | 2.66 | 2.02 | 289 | N.A. | N.A. | 16.41 | 31.77 | I | 5 |
| P30 | 10 | M | No | 26 | 24 | S | No | 138.3 | -0.3 | 0.520 | -1.7 | 3 | 1 | - | - | - | - | - | - | - | 2.55 | 1.31 | 248 | 805.7 | 124.3 | 18.23 | 13.21 | IV | 4 |
| P31 | 30.5 | F | No | 30 | 26 | S | No | 159.2 | -0.3 | 0.955 | -1.3 | 5 | 1 | - | - | + | - | + | - | + | 2.48 | 1.08 | 62 | 295.7 | 28.43 | 59.77 | 13.51 | I | 5 |
| P32 | 11.5 | F | No | 29 | 26 | S | one unaffected brother | 144.2 | -0.8 | 0.706 | -0.4 | 5 | 1 | - | - | - | - | + | + | - | 2.37 | 1.51 | 470 | 1674 | 196.1 | 35.43 | 19.58 | I | 4 |
| P33 | 4 | F | No | 29 | 28 | S | No | 90.8 | -3.2 | 0.418 | -4.3 | 2 | 2 | - | - | - | + | - | - | - | 2.61 | 1.66 | 153 | 642.2 | 32.16 | 15.26 | 54.81 | IV | 12 |
| P34 | 22.7 | M | No | 27 | 27 | S | one unaffected brother | 161.5 | -1.8 | 0.744 | -3.2 | 6 | 1 | - | - | - | - | + | - | - | 2.34 | 1.15 | 79 | 364.7 | 25.25 | 39.69 | 15.59 | I | 6 |
| P35 | 1.1 | F | No | N.A. | 28 | S | No | N.A. | N.A. | N.A. | N.A. | N.A. | N.A. | N.A. | N.A. | N.A. | N.A. | N.A. | N.A. | N.A. | N.A. | N.A. | N.A. | N.A. | N.A. | N.A. | N.A. | N.A. | N.A. |
| P36 | 2 | M | No | 27 | 27 | MAR | No | 93.5 | 1.5 | 0.343 | -1.6 | 3 | 2 | - | - | - | - | + | - | - | 2.45 | 1.6 | 316 | 1494 | 164 | 40.64 | 54.03 | I | 5 |
| P37 | 5 | F | No | 26 | 25 | S | One affected twin sister | N.A. | N.A. | N.A. | N.A. | 3 | 1 | - | - | - | - | - | + | - | N.A. | N.A. | N.A. | N.A. | N.A. | N.A. | N.A. | IV | N.A. |
| P38 | 24 | F | No | 28 | 26 | S | No | N.A. | N.A. | N.A. | N.A. | 8 | 1 | - | + | - | - | - | + | + | N.A. | N.A. | N.A. | N.A. | N.A. | N.A. | N.A. | III | N.A. |
| P39 | 17 | M | No | N.A. | N.A. | S | No | N.A. | N.A. | 0.584 | -3.3 | N.A. | N.A. | N.A. | N.A. | N.A. | N.A. | N.A. | N.A. | N.A. | N.A. | N.A. | N.A. | N.A. | N.A. | N.A. | N.A. | N.A. | N.A. |
| P40 | 27 | F | No | 37 | N.A. | S | No | 150.3 | -1.9 | 0.882 | -1 | 3 | 1 | - | - | - | - | + | - | - | 2.3 | 1.03 | 88 | 99 | 33 | 40 | 22 | I | 4 |
| P41 | 5 | M | No | 25 | 25 | S | No | N.A. | N.A. | N.A. | N.A. | 6 | 1 | + | - | - | - | + | + | - | N.A. | N.A. | N.A. | N.A. | N.A. | N.A. | N.A. | I | N.A. |
| P42 | 12 | M | No | 23 | 22 | S | No | N.A. | N.A. | N.A. | N.A. | N.A. | N.A. | N.A. | N.A. | - | - | + | - | - | 2.41 | 0.97 | 85 | N.A. | N.A. | 54.93 | 7.84 | N.A. | N.A. |
| P43 | 26.3 | M | No | 24 | 26 | S | Two unaffected brothers | 145 | -4.5 | 0.709 | -2.8 | 100+ | 5 | - | + | - | + | - | + | - | N.A. | N.A. | N.A. | N.A. | N.A. | N.A. | N.A. | III | 18 |
| P44 | 20.6 | M | No | 27 | 25 | S | No | 145 | -4.5 | 0.647 | -3.7 | 8 | 1 | - | + | - | + | + | + | - | 2.5 | 1.5 | 317 | 902 | 200.9 | 45.21 | 21.35 | III | 15 |
| P45 | 3.3 | M | No | N.A. | N.A. | S | No | 87 | -3.2 | 0.385 | -4.3 | 3 | 1 | - | - | - | - | + | - | - | N.A. | N.A. | N.A. | N.A. | N.A. | N.A. | N.A. | I | 9 |
| P46 | 14 | M | No | 31 | 26 | S | No | 160 | -0.8 | 0.373 | -3.8 | 3 | 1 | - | + | + | - | - | - | - | 2.44 | 1.01 | 71 | 442.9 | 21.62 | 18.76 | 38.1 | III | 11 |
| P47 | 11 | M | No | 26 | 18 | S | No | 135 | -1.6 | N.A. | N.A. | 5 | 1 | - | - | - | - | + | + | - | N.A. | N.A. | N.A. | N.A. | N.A. | N.A. | N.A. | I | N.A. |
| P48 | 16 | M | No | 39 | 34 | S | one unaffected brother | 155.4 | -2.6 | N.A. | -2.0 | 4 | 2 | - | + | - | + | + | - | - | N.A. | N.A. | N.A. | N.A. | N.A. | N.A. | N.A. | III | 12 |
| P49 | 2 | F | No | 27 | 29 | S | No | 75 | -3.6 | N.A. | N.A. | 2 | 1 | - | + | - | - | + | - | - | N.A. | N.A. | N.A. | N.A. | N.A. | N.A. | N.A. | III | N.A. |
| P50 | 0.2 | M | No | 27 | 27 | S | No | N.A. | N.A. | N.A. | N.A. | 3 | 3 | - | - | N.A. | N.A. | N.A. | N.A. | N.A. | N.A. | N.A. | N.A. | N.A. | N.A. | N.A. | N.A. | N.A. | N.A. |
| P51 | 25.6 | F | No | 42 | 26 | S | No | 118 | -7.9 | 0.998 | -1.0 | 5 | 1 | - | + | - | + | - | + | - | 2.3 | 1.08 | 52 | 235.6 | 14.46 | 51.53 | 11.01 | III | 13 |

P, proband; M, male; F, female; N/A, not available; SDS, standard deviation score; LS-BMD, lumbar spine-bone mineral density; BS, blue sclera; DI, dentinogenesis imperfecta; HL, hearing loss; S, spontaneous conception; MAR, medical assisted conception; Ca, calcium (mmol/L); P, phosphate (mmol/L); ALP, alkaline phosphatase (U/L); β-CTX, beta cross-linked C-terminal telopeptide of type 1 collagen (ng/L); OC, osteocalcin (ng/mL); PTH, intact parathyroid hormone (pg/mL); 25OHD, 25-hydroxyvitamin D (ng/mL); “+” indicates that the patient has the symptom; “-” indicates that the patient does not have the symptoms.
