# Supplementary material for: Comparing Clinical and Genetic Characteristics of De Novo and Inherited COL1A1/COL1A2 Variants in a Large Chinese Cohort of Osteogenesis Imperfecta
Source: Front Endocrinol (Lausanne). 2022 Jul 14;13:935905. doi: 10.3389/fendo.2022.935905 (PMC9329653; doi:10.3389/fendo.2022.935905)
Supplement: Supplementary Figure 1 — De novo and inherited mutation spectrum of COL1A1/COL1A2. (A) De novo mutation spectrum of COL1A1. (B) De novo mutation spectrum of COL1A2. (C) Inherited mutation spectrum of COL1A1. (D) Inherited mutation spectrum of COL1A2. [file DataSheet_1.zip › Supplementary material/Supplementary Table 2.docx]

**SUPPLEMENTARY TABLE 2┃**Mutations in *COL1A1/COL1A2* of probands with *de novo* mutations

| **Gene** | **Proband ID** | **Exon or intron** | **Nucleotide change** | **Amino acid change** | **Mutation effect** | **Parental gene verification** |
| --- | --- | --- | --- | --- | --- | --- |
| *COL1A1* | P1 | Exon 32 | c.2161C>T | p.Gln721* | Nonsense | f/m/b |
|  | P2 | Exon 8 | c.635G>A | p.Gly212Glu | Missense | f/m |
|  | P3 | Exon 47 | c.3521C>T | p.Ala1174Val | Missense | f/m/s |
|  | P4 | Exon 8 | c.607G>A | p.Gly203Ser | Missense | f/m/b1/b2 |
|  | P5 | Exon 36 | c.2461G>A | p.Gly821Ser | Missense | f/m/s |
|  | P6 | Exon 17 | c.1111G>A | p.Gly371Ser | Missense | f/m |
|  | P7 | Exon 17 | c.1081C>T | Arg361* | Nonsense | f/m |
|  | P8 | Intron 12 | c.859-2A>C | / | Splice | f/m |
|  | P9 | Exon 44 | c.3226G>A | p.Gly1076Ser | Missense | f/m |
|  | P10 | Exon 32 | c.2155G>A | p.Gly719Ser | Missense | f/m |
|  | P11 | Intron 25 | c.1768-1G>A | / | Splice | f/m |
|  | P12 | Exon 5 | c.433_434insC | p.Gly145Alafs*24 | Frameshift | f/m |
|  | P13 | Exon 17 | c.1121G>C | p.Gly374Ala | Missense | f/m |
|  | P14 | Exon 40 | c.2867G>C | p.Gly956Ala | Missense | f/m |
|  | P15 | Exon 49 | c.3893C>T | p.Thr1298Ile | Missense | f/m |
|  | P16 | Exon 17 | c.1121delG | p.Gly374Alafs*167 | Frameshift | f/m |
|  | P17 | Exon 48 | c.3638delG | p.Gly1213Alafs*26 | Frameshift | f/m |
|  | P18 | Exon 33 | c.2297C>G | p.Thr766Ser | Missense | f/m/s |
|  | P19 | Exon 42 | c.3076C>T | p.Arg1026* | Nonsense | f/m |
|  | P20 | Exon 39 | c.2775delT | p.Gly926Valfs*182 | Frameshift | f/m |
|  | P21 | Exon 7 | c.569delC | p.Pro190Leufs*75 | Frameshift | f/m |
|  | P22 | Exon 35 | c.2410G>T | p.Glu804* | Nonsense | f/m |
|  | P23 | Exon 17 | c.1128delT | p.Gly377Alafs*164 | Frameshift | f/m |
|  | P24 | Exon 32 | c.2183G>A | p.Gly728Glu | Missense | f/m |
|  | P25 | Exon 5 | c.441dupC | p.Gly148Argfs*21 | Frameshift | f/m |
|  | P26 | Exon 33 | c.2263G>A | p.Gly755Ser | Missense | f/m |
|  | P27 | Exon 48 | c.3647A>G | p.Tyr1216Cys | Missense | f/m |
|  | P28 | Exon 23 | c.1594delG | p.Ala532Leufs*9 | Frameshift | f/m |
|  | P29 | Intron 19 | c.1299+1G>C | / | Splice | f/m/b |
|  | P30 | Exon 31 | c.2089C>T | p.Arg697* | Nonsense | f/m |
|  | P31 | Intron 26 | c.1821+1G>A | / | Splice | f/m |
|  | P32 | Exon 47 | c.3431dupC | p.Gly1145Trpfs*29 | Frameshift | f/m |
|  | P33 | Exon 44 | c.3226G>A | p.Gly1076Ser | Missense | f/m |
|  | P34 | Intron 2 | c.299-1G>A | / | Splice | f/m |
|  | P35 | Exon 39 | c.2669G>C | p.Gly890Ala | Missense | f/m |
|  | P36 | Exon 2 | c.285C>A | p.Cys95* | Nonsense | f/m |
|  | P37 | Exon 34 | c.2362G＞A | p.Gly788Ser | Missense | f/m/ts |
|  | P38 | Exon 8 | c.607G>A | p.Gly203Ser | Missense | f/m |
|  | P39 | Exon 2 | c.182G>T | p.Cys61Phe | Missense | f/m |
|  | P40 | Exon 7 | c.588+2T>A | / | Splice | f/m |
|  | P41 | Intron 12 | c.858+1_858+5del | / | Splice | f/m |
| *COL1A2* | P42 | Exon 12 | c.586G>T | p.Gly196Cys | Missense | f/m |
|  | P43 | Exon 38 | c.2332G>A | p.Gly778Ser | Missense | f/m/b1/b2 |
|  | P44 | Exon 35 | c.2081G>A | p.Gly694Asp | Missense | f/m |
|  | P45 | Exon 49 | c.3304G>A | p.Gly1102Ser | Missense | f/m |
|  | P46 | Exon 34 | c.2035G>A | p.Gly679Ser | Missense | f/m |
|  | P47 | Exon 48 | c.3197G>T | p.Gly1066Val | Missense | f/m |
|  | P48 | Exon 48 | c.3250G>T | p.Gly1084Cys | Missense | f/m |
|  | P49 | Exon 51 | c.3815G>T | p.Cys1272Phe | Missense | f/m |
|  | P50 | Exon 17 | c.838G>C | p.Gly280Arg | Missense | f/m |
|  | P51 | Exon 37 | c.2279G>A | p.Gly760Glu | Missense | f/m |

P, proband; f, father; m, mother; b, brother; s, sister; ts, twin sister.
