# Supplementary material for: Comparing Clinical and Genetic Characteristics of De Novo and Inherited COL1A1/COL1A2 Variants in a Large Chinese Cohort of Osteogenesis Imperfecta
Source: Front Endocrinol (Lausanne). 2022 Jul 14;13:935905. doi: 10.3389/fendo.2022.935905 (PMC9329653; doi:10.3389/fendo.2022.935905)
Supplement: Supplementary Figure 1 — De novo and inherited mutation spectrum of COL1A1/COL1A2. (A) De novo mutation spectrum of COL1A1. (B) De novo mutation spectrum of COL1A2. (C) Inherited mutation spectrum of COL1A1. (D) Inherited mutation spectrum of COL1A2. [file DataSheet_1.zip › Supplementary material/Supplementary Table 3.docx]

**SUPPLEMENTARY TABLE 3┃**Clinical characteristics of probands with inherited mutations

| **Proband ID** | **Age at diagnosis** | **Sex** | **Source** | **Height(cm)** | **SDS** | **BMD** | | **Fractures** | | **Bowing of lower limbs** | | **Scoliosis** | | **BS** | **DI** | **HL** | **Ca** | **P** | **ALP** | **β-CTX** | **OC** | **PTH** | **25OHD** | **Clinical type** | **Clinical score** |
| --- | --- | --- | --- | --- | --- | --- | --- | --- | --- | --- | --- | --- | --- | --- | --- | --- | --- | --- | --- | --- | --- | --- | --- | --- | --- |
|  |  |  |  |  |  | **LS-BMD (g/cm2)** | **Z/T-score** | **Total** | **Frequency (per year)** | **Mild** | **Severe** | **Mild** | **Severe** |  |  |  |  |  |  |  |  |  |  |  |  |
| p1 | 22 | F | m | 155 | -1 | 1.178 | 0.6 | 7 | 1 | - | - | - | - | + | - | + | N.A. | N.A. | N.A. | N.A. | N.A. | N.A. | N.A. | I | 4 |
| p2 | 29 | M | f | 162 | -1.8 | 0.744 | -3.2 | 20+ | 1 | - | + | - | - | + | + | - | 2.33 | 1.2 | 86 | 1768 | 102 | 88 | 11 | III | 11 |
| p3 | 26 | M | m | 164.5 | -1.3 | 1.139 | -0.2 | 2 | 1 | - | - | - | - | + | - | - | N.A. | N.A. | N.A. | N.A. | N.A. | N.A. | N.A. | I | 4 |
| p4 | 22 | M | f | 144 | -4.7 | 0.711 | -2.2 | 2 | 1 | - | - | + | - | + | - | + | N.A. | N.A. | N.A. | N.A. | N.A. | N.A. | N.A. | I | 10 |
| p5 | 22 | M | f | 147 | -4.2 | N.A. | N.A. | 20+ | 2 | - | + | - | - | - | - | - | 2.46 | 1.34 | 368 | 1117 | 120.4 | 73.1 | 27.14 | III | N.A. |
| p6 | 14.8 | M | m | 156 | -2.0 | 0.695 | -1.3 | 3 | 1 | - | - | - | - | + | - | - | 2.21 | 1.1 | 152 | 357 | 31.2 | 68 | 14.7 | I | 4 |
| p7 | 4 | F | f | N.A. | N.A. | N.A. | N.A. | 6 | 2 | - | + | - | - | + | + | - | N.A. | N.A. | N.A. | N.A. | N.A. | N.A. | N.A. | III | N.A. |
| p8 | 28 | F | f | N.A. | N.A. | N.A. | N.A. | N.A. | N.A. | N.A. | N.A. | N.A. | N.A. | N.A. | N.A. | N.A. | N.A. | N.A. | N.A. | N.A. | N.A. | N.A. | N.A. | N.A. | N.A. |
| p9 | 10.6 | M | m | 144 | 0.1 | 0.532 | -1.8 | 8 | 1 | - | - | - | - | + | + | - | N.A. | N.A. | N.A. | N.A. | N.A. | N.A. | N.A. | I | 4 |
| p10 | 3.5 | M | m | 93.5 | -1.8 | 0.375 | -4.5 | 4 | 2 | - | - | - | - | + | - | - | N.A. | N.A. | N.A. | N.A. | N.A. | N.A. | N.A. | I | 8 |
| p11 | 8.9 | F | m | 115.5 | -3.4 | 0.439 | -2.5 | 4 | 1 | - | - | - | - | + | - | - | N.A. | N.A. | N.A. | N.A. | N.A. | N.A. | N.A. | I | 7 |
| p12 | 10.8 | M | m | 135.5 | -1.4 | 0.56 | -1.4 | 4 | 1 | - | - | - | - | + | + | - | N.A. | N.A. | N.A. | N.A. | N.A. | N.A. | N.A. | I | 4 |
| p13 | 14.3 | M | f | 166 | -0.2 | 0.629 | -1.1 | N.A. | N.A. | - | - | - | - | + | - | - | N.A. | N.A. | N.A. | N.A. | N.A. | N.A. | N.A. | I | N.A. |
| p14 | 35 | M | m | 165 | -1.3 | 0.846 | -2.6 | 9 | 1 | - | - | - | - | + | + | + | N.A. | N.A. | N.A. | N.A. | N.A. | N.A. | N.A. | I | 5 |
| p15 | 44.5 | M | f | 155 | -2.9 | 0.676 | -4.7 | 8 | 1 | - | - | - | - | + | + | - | N.A. | N.A. | N.A. | N.A. | N.A. | N.A. | N.A. | I | 8 |
| p16 | 11.3 | F | f | 160 | 1.8 | 0.723 | -0.2 | 8 | 1 | - | - | - | - | + | + | - | N.A. | N.A. | N.A. | N.A. | N.A. | N.A. | N.A. | I | 4 |
| p17 | 10.6 | M | f | 140 | -0.5 | 0.528 | -1.6 | 6 | 1 | - | - | - | - | - | - | - | 2.65 | 1.66 | 235 | N.A. | N.A. | N.A. | N.A. | IV | 4 |
| p18 | 23.9 | F | f | 146.5 | -2.6 | 0.796 | -1.8 | 7 | 1 | - | - | - | - | + | - | - | 2.24 | 1.09 | 60 | N.A. | N.A. | 41.03 | 29.17 | I | 5 |
| p19 | 7.6 | M | f | 134.5 | 1.3 | 0.49 | -1.7 | 7 | 1 | - | - | + | - | + | - | - | N.A. | N.A. | 92 | N.A. | N.A. | N.A. | N.A. | I | 5 |
| p20 | 18.8 | M | f | 167 | -0.9 | 0.934 | -0.4 | 11 | 1 | - | - | - | - | + | - | - | N.A. | N.A. | N.A. | N.A. | N.A. | N.A. | N.A. | I | 5 |
| p21 | 18.3 | M | m | 163 | -1.6 | 0.992 | 0.1 | 20+ | 2 | - | - | - | - | + | - | - | N.A. | N.A. | N.A. | N.A. | N.A. | N.A. | N.A. | I | 6 |
| p22 | 8.5 | M | m | 112.5 | -3.7 | 0.44 | -2.5 | 8 | 1 | - | - | + | - | - | + | - | N.A. | N.A. | N.A. | N.A. | N.A. | N.A. | N.A. | IV | 8 |
| p23 | 2 | M | m | 84 | -1.3 | N.A. | N.A. | 5 | 1 | - | - | - | - | + | - | - | N.A. | N.A. | N.A. | N.A. | N.A. | N.A. | N.A. | I | N.A. |
| p24 | 4.5 | M | f | 123.6 | 3.9 | 0.733 | 4.2 | 6 | 2 | - | - | - | - | - | - | - | 2.57 | 1.62 | 407 | 1459 | 95.9 | 71.54 | 8.16 | IV | 5 |
| p25 | 12 | M | f | 152 | 0.0 | 0.71 | -0.2 | 4 | 1 | - | - | - | - | + | - | - | N.A. | N.A. | N.A. | N.A. | N.A. | N.A. | N.A. | I | 4 |
| p26 | 2.3 | M | m | 87 | -1.3 | N.A. | N.A. | 4 | 1 | - | - | - | - | + | - | - | 2.43 | 1.49 | 398 | 968.5 | 181.8 | 24.21 | 23.75 | I | N.A. |
| p27 | 14.7 | F | m | 162.6 | 0.5 | 0.811 | -1.2 | 5 | 1 | - | - | + | - | + | - | - | N.A. | N.A. | N.A. | N.A. | N.A. | N.A. | N.A. | I | 5 |
| p28 | 23.1 | F | m | 156.7 | -0.7 | 1.101 | 0.1 | 1 | 1 | - | - | - | - | - | - | - | N.A. | N.A. | N.A. | N.A. | N.A. | N.A. | N.A. | IV | 4 |
| p29 | 11.8 | M | f | 150 | -0.1 | 0.471 | -2.4 | 2 | 1 | - | - | - | - | + | - | - | N.A. | N.A. | N.A. | N.A. | N.A. | N.A. | N.A. | I | 5 |
| p30 | 8.9 | M | f | 133.6 | -0.2 | 0.568 | -0.5 | 4 | 1 | - | - | - | - | + | + | - | N.A. | N.A. | N.A. | N.A. | N.A. | N.A. | N.A. | I | 4 |
| p31 | 13.8 | M | m | 154.8 | -1.4 | 0.61 | -1.6 | 5 | 2 | - | - | - | - | + | - | - | N.A. | N.A. | N.A. | N.A. | N.A. | N.A. | N.A. | I | 5 |
| p32 | 10.9 | M | f | 137.2 | -1.2 | 0.638 | -0.4 | 3 | 1 | - | - | - | - | + | - | - | N.A. | N.A. | N.A. | N.A. | N.A. | N.A. | N.A. | I | 4 |
| p33 | 7 | M | m | N.A. | N.A. | N.A. | N.A. | N.A. | N.A. | N.A. | N.A. | N.A. | N.A. | N.A. | N.A. | N.A. | N.A. | N.A. | N.A. | N.A. | N.A. | N.A. | N.A. | N.A. | N.A. |
| p34 | 12 | F | m | 134 | -2.8 | 0.652 | -1.7 | 2 | 1 | - | - | - | - | + | - | - | N.A. | N.A. | N.A. | N.A. | N.A. | N.A. | N.A. | I | 5 |
| p35 | 8.9 | M | m | 127.4 | -1.3 | 0.382 | -2.6 | 3 | 1 | - | - | - | - | + | - | - | N.A. | N.A. | N.A. | N.A. | N.A. | N.A. | N.A. | I | 5 |
| p36 | 9 | M | m | 139 | 0.6 | N.A. | N.A. | 7 | 2 | - | - | - | - | - | - | - | N.A. | N.A. | N.A. | N.A. | N.A. | N.A. | N.A. | IV | N.A. |
| p37 | 5.7 | F | f | 110.2 | -1.0 | 0.522 | 0.4 | 4 | 1 | - | - | - | - | + | - | - | 2.64 | 1.84 | 247 | 683.9 | 59.73 | 15.7 | 43.77 | I | 4 |
| p38 | 13 | M | m | N.A. | N.A. | N.A. | N.A. | N.A. | N.A. | N.A. | N.A. | N.A. | N.A. | N.A. | N.A. | N.A. | N.A. | N.A. | N.A. | N.A. | N.A. | N.A. | N.A. | N.A. | N.A. |
| p39 | 7.8 | F | f | 129.1 | 0.3 | 0.608 | 0.0 | 2 | 1 | - | - | - | - | + | - | - | N.A. | N.A. | N.A. | N.A. | N.A. | N.A. | N.A. | I | 4 |
| p40 | 1.3 | M | f | 86 | 2.2 | N.A. | N.A. | 2 | 2 | - | - | - | - | - | - | - | 2.74 | 1.67 | 588 | N.A. | N.A. | 27.62 | 66.53 | IV | N.A. |
| p41 | 4 | F | f | 122 | 4.8 | N.A. | N.A. | 2 | 2 | - | - | - | - | + | - | - | N.A. | N.A. | N.A. | N.A. | N.A. | N.A. | N.A. | I | N.A. |
| p42 | 12.8 | M | f | 143 | -2.0 | 0.389 | -4.0 | 3 | 1 | - | - | + | - | - | - | - | 2.43 | 1.69 | 213 | 1272 | 57.43 | 18.68 | 52.65 | IV | 7 |
| p43 | 24 | M | m | 182 | 1.5 | 0.646 | -2.8 | 1 | 1 | - | - | - | - | + | + | - | N.A. | N.A. | N.A. | N.A. | N.A. | N.A. | N.A. | I | 5 |
| p44 | 28.6 | M | m | 158.3 | -2.4 | 1.04 | -0.4 | 16 | 1 | - | - | - | - | - | + | - | 2.36 | 1.05 | 112 | 489.7 | 17.74 | 29.24 | 22.73 | IV | 6 |
| p45 | 17.5 | M | f | 166.4 | -1.0 | 0.808 | -1.4 | 5 | 1 | - | - | + | - | + | + | - | 2.34 | 1.43 | 195 | 1166 | 120.1 | 26.43 | 18.63 | I | 5 |
| p46 | 34 | M | f | 172.4 | 0.0 | 0.851 | -2.2 | 6 | 1 | - | - | - | - | + | - | - | 2.36 | 1.15 | 103 | 407.6 | 40.24 | 53.67 | 28 | I | 5 |
| p47 | 30 | F | m | 150.7 | -1.8 | 0.655 | -3.6 | 4 | 1 | - | - | - | - | + | - | + | 2.6 | 1.27 | 109 | 509.4 | 63.98 | 30.02 | 20.67 | I | 6 |
| p48 | 36 | F | m | 157.6 | -0.6 | 0.996 | -1.0 | 10+ | 1 | - | - | - | - | + | + | - | 2.21 | 1.01 | 46 | N.A. | N.A. | 25.55 | 21.28 | I | 5 |
| p49 | 33 | M | m | 155 | -2.9 | 0.975 | -1.1 | 12 | 1 | - | - | - | - | + | + | + | 2.43 | 1.09 | 131 | 382.3 | 19.69 | 55.13 | 19.13 | I | 6 |
| p50 | 38 | F | f | 147 | -2.5 | 0.796 | -2.1 | 8 | 1 | - | - | - | - | + | - | - | 2.27 | 1.21 | 101 | 185.7 | 18.28 | 45.69 | 16.86 | I | 6 |
| p51 | 26.5 | F | f | 152.7 | -1.5 | 0.888 | -1.9 | 4 | 1 | - | - | + | - | + | - | + | 2.41 | 1.32 | 100 | 531.5 | 45.91 | 33.73 | 17.68 | I | 5 |
| p52 | 40 | F | m | 159 | -0.3 | 0.692 | -3.0 | 27 | 1 | - | - | - | - | + | - | - | 2.3 | 1.06 | 52 | N.A. | N.A. | 58.01 | 28.99 | I | 6 |
| p53 | 14 | M | f | 157.2 | -1.2 | 0.871 | 3.7 | 7 | 2 | - | - | - | - | + | - | - | 2.51 | 1.36 | 213 | 857.4 | 146.8 | 22.46 | 18.34 | I | 7 |
| p54 | 3.9 | M | f | 94.5 | -2.3 | 0.314 | -5.7 | 4 | 4 | - | - | - | - | + | - | - | N.A. | N.A. | N.A. | N.A. | N.A. | N.A. | N.A. | I | 10 |
| p55 | 2.5 | F | f | 80 | -3.3 | N.A. | N.A. | 4 | 2 | - | - | - | - | + | - | - | 2.39 | 0.83 | 618 | 143 | 12.29 | 53.62 | N.A. | I | N.A. |
| p56 | 2 | M | f | 70 | -5.4 | N.A. | N.A. | 6 | 3 | - | + | - | - | + | - | - | 2.43 | 1.12 | 134 | 655 | 77.48 | 50.8 | 14.71 | III | N.A. |
| p57 | 27 | M | f | 162 | -1.8 | 0.814 | -1.5 | 50+ | 5 | - | - | - | - | - | - | - | N.A. | N.A. | N.A. | N.A. | N.A. | N.A. | N.A. | IV | 8 |
| p58 | 9.8 | M | m | 124 | -2.5 | 0.492 | -2.1 | 18 | 3 | - | - | - | - | + | - | - | N.A. | N.A. | N.A. | N.A. | N.A. | N.A. | N.A. | I | 8 |
| p59 | 17 | M | f | N.A. | N.A. | N.A. | N.A. | 3 | 2 | - | - | - | - | - | - | - | 2.42 | 1.39 | 224 | N.A. | N.A. | 57.96 | 16.06 | IV | N.A. |
| p60 | 5 | M | f | 93 | -4.3 | N.A. | N.A. | 6 | 2 | + | - | - | - | + | + | - | N.A. | N.A. | N.A. | N.A. | N.A. | N.A. | N.A. | I | N.A. |
| p61 | 9 | F | m | 130 | -0.7 | N.A. | N.A. | 5 | 1 | - | + | - | - | + | - | - | N.A. | N.A. | N.A. | N.A. | N.A. | N.A. | N.A. | III | N.A. |
| p62 | 8 | M | f | N.A. | N.A. | N.A. | N.A. | N.A. | N.A. | N.A. | N.A. | N.A. | N.A. | N.A. | N.A. | N.A. | N.A. | N.A. | N.A. | N.A. | N.A. | N.A. | N.A. | N.A. | N.A. |
| p63 | 11 | F | m | 125 | -3.3 | N.A. | N.A. | N.A. | N.A. | N.A. | N.A. | N.A. | N.A. | N.A. | N.A. | N.A. | N.A. | N.A. | N.A. | N.A. | N.A. | N.A. | N.A. | N.A. | N.A. |
| p64 | 7.6 | M | m | 116.5 | -2.1 | 0.409 | -2.9 | 2 | 1 | - | - | - | - | + | - | - | N.A. | N.A. | N.A. | N.A. | N.A. | N.A. | N.A. | I | 6 |
| p65 | 6.3 | M | m | 113 | -1.4 | 0.353 | -3.6 | 4 | 1 | - | - | - | - | + | - | - | 2.27 | 0.96 | 168 | N.A. | N.A. | 61.05 | 20.8 | I | 6 |
| p66 | 8.8 | F | m | 122 | -2.0 | 0.474 | -2.1 | 3 | 1 | - | - | - | - | + | - | - | N.A. | N.A. | N.A. | N.A. | N.A. | N.A. | N.A. | I | 5 |
| p67 | 6 | M | f | N.A. | N.A. | N.A. | N.A. | N.A. | N.A. | N.A. | N.A. | N.A. | N.A. | N.A. | N.A. | N.A. | N.A. | N.A. | N.A. | N.A. | N.A. | N.A. | N.A. | N.A. | N.A. |
| p68 | 25.1 | M | m | 136.5 | -5.9 | 0.774 | -2.4 | 8 | 1 | - | + | - | - | + | + | - | N.A. | N.A. | N.A. | N.A. | N.A. | N.A. | N.A. | III | 12 |
| p69 | 8 | F | f | 120 | -1.6 | N.A. | N.A. | 5 | 1 | - | - | - | - | - | - | - | N.A. | N.A. | N.A. | N.A. | N.A. | N.A. | N.A. | IV | N.A. |
| p70 | 6 | M | f | N.A. | N.A. | N.A. | N.A. | N.A. | N.A. | - | - | - | - | N.A. | N.A. | N.A. | N.A. | N.A. | N.A. | N.A. | N.A. | N.A. | N.A. | N.A. | N.A. |
| p71 | 19.9 | M | f | 155.4 | -2.8 | 0.94 | -0.3 | 2 | 1 | - | - | - | - | - | - | - | N.A. | N.A. | N.A. | N.A. | N.A. | N.A. | N.A. | IV | 5 |
| p72 | 7.8 | F | m | 113.1 | -2.7 | 0.339 | -3.3 | 8 | 2 | - | - | - | - | - | - | - | N.A. | N.A. | N.A. | N.A. | N.A. | N.A. | N.A. | IV | 8 |
| p73 | 6.9 | M | f | 113.3 | -2.0 | 0.356 | -3.6 | 5 | 1 | - | - | - | - | + | + | - | N.A. | N.A. | N.A. | N.A. | N.A. | N.A. | N.A. | I | 6 |
| p74 | 9.1 | F | f | 126.5 | -1.4 | 0.628 | -0.1 | 3 | 1 | - | - | - | - | - | - | - | N.A. | 1.59 | N.A. | 335 | N.A. | N.A. | N.A. | IV | 4 |
| p75 | 3.4 | M | f | 97.4 | -0.8 | 0.361 | -5.6 | 2 | 1 | - | - | - | - | + | + | - | 2.42 | 1.73 | 274 | 835.9 | 66.35 | 25.99 | 34.31 | I | 7 |
| p76 | 17.8 | F | f | 134 | -4.9 | 0.879 | -0.8 | 5 | 1 | - | - | + | - | + | + | - | 2.39 | 1.7 | 117 | 260 | 29.41 | 29.18 | 18.61 | I | 8 |
| p77 | 10.5 | M | m | 133.8 | -2.1 | 0.37 | -3.8 | 7 | 2 | + | - | + | - | + | + | - | 2.55 | 1.5 | 221 | 608.5 | 61.61 | 20.59 | 30.74 | I | 11 |
| p78 | 9.4 | M | m | 126.7 | -1.8 | 0.484 | -1.5 | 4 | 1 | - | - | - | - | + | + | - | 2.31 | 1.67 | 245 | 1223 | 106.5 | 51.43 | 24.51 | I | 4 |
| p79 | 11.5 | M | f | 157.1 | 1.2 | 0.441 | -2.9 | 5 | 1 | + | - | - | - | - | + | - | 2.49 | 1.33 | 265 | 736.6 | 49.95 | 29.07 | 31.27 | IV | 5 |
| p80 | 15.7 | F | f | 151.4 | -1.6 | 0.777 | -2.1 | 4 | 1 | - | - | - | - | + | + | - | 2.28 | 1.04 | 277 | 1393 | 87.54 | 44.82 | 15.88 | I | 5 |
| p81 | 10.8 | M | m | 141.8 | -0.4 | 0.619 | 0.1 | 4 | 1 | - | - | - | - | + | + | - | 2.33 | 1.8 | 359 | 980 | 67 | 35 | 25 | I | 4 |
| p82 | 5.1 | M | f | 112.9 | 0.2 | 0.442 | 0.0 | 4 | 3 | - | - | - | - | - | - | - | 2.4 | 1.45 | 325 | 858.4 | 40.81 | 47.17 | 47.86 | IV | 5 |
| p83 | 17.8 | F | m | 147 | -2.5 | 0.918 | -1.2 | 4 | 1 | - | - | - | - | + | - | - | 2.45 | 1.36 | 68 | 889.6 | 42.13 | 37.33 | 15.44 | I | 5 |
| p84 | 4.4 | M | f | 102.9 | -1.0 | 0.410 | -1.9 | 1 | 1 | - | - | - | - | + | - | - | 2.53 | 1.5 | 293 | 1407 | 70.22 | 37.91 | 30.7 | I | 4 |

p, proband; M, male; F, female; f, father; m, mother; N/A, not available; SDS, standard deviation score; LS-BMD, lumbar spine-bone mineral density; BS, blue sclera; DI, dentinogenesis imperfecta; HL, hearing loss; “+” indicates that the patient has the symptom; Ca, calcium (mmol/L); P, phosphate (mmol/L); ALP, alkaline phosphatase (U/L); β-CTX, beta cross-linked C-terminal telopeptide of type 1 collagen (ng/L); OC, osteocalcin (ng/mL); PTH, intact parathyroid hormone (pg/mL); 25OHD, 25-hydroxyvitamin D (ng/mL); “-” indicates that the patient does not have the symptoms.
