# Supplementary material for: Comparing Clinical and Genetic Characteristics of De Novo and Inherited COL1A1/COL1A2 Variants in a Large Chinese Cohort of Osteogenesis Imperfecta
Source: Front Endocrinol (Lausanne). 2022 Jul 14;13:935905. doi: 10.3389/fendo.2022.935905 (PMC9329653; doi:10.3389/fendo.2022.935905)
Supplement: Supplementary Figure 1 — De novo and inherited mutation spectrum of COL1A1/COL1A2. (A) De novo mutation spectrum of COL1A1. (B) De novo mutation spectrum of COL1A2. (C) Inherited mutation spectrum of COL1A1. (D) Inherited mutation spectrum of COL1A2. [file DataSheet_1.zip › Supplementary material/Supplementary Table 4.docx]

**SUPPLEMENTARY TABLE 4**┃Mutations in *COL1A1/COL1A2* of probands with inherited mutations

| **Gene** | **Proband ID** | **Exon or intron** | **Nucleotide change** | **Amino acid change** | **Mutation effect** | **Source** |
| --- | --- | --- | --- | --- | --- | --- |
| *COL1A1* | p1 | Exon 28 | c.1893_1894delAG | p.Gly632* | Nonsense | m* |
|  | p2 | Exon 50 | c.4021C>T | p.Gln1341* | Nonsense | f* |
|  | p3 | Exon 9 | c.669delA | p.Gly224Valfs*41 | Frameshift | m* |
|  | p4 | Exon 11 | c.769G>A | p.Gly257Arg | Missense | f*# |
|  | p5 | Exon 36 | c.2461G>A | p.Gly821Ser | Missense | f* |
|  | p6 | Exon 44 | c.3235G>A | p.Gly1079Ser | Missense | m* |
|  | p7 | Exon 33 | c.2335G>A | p.Gly779Ser | Missense | f* |
|  | p8 | Exon 42 | c.3076C>T | p.Arg1026* | Nonsense | f* |
|  | p9 | Exon 9 | c.658C>T | p.Phe220* | Nonsense | m* |
|  | p10 | Intron 36 | c.2559+1G>A | / | Splice | m* |
|  | p11 | Exon 45 | c.3349C>T | p.Gln1117* | Nonsense | m* |
|  | p12 | Exon 35 | c.2424delC | p.Gly809Alafs*299 | Frameshift | m* |
|  | p13 | Exon 32 | c.2235_2235+1delAG | / | Splice | f*# |
|  | p14 | Intron 4 | c.370-1G>A | / | Splice | m* |
|  | p15 | Exon 11 | c.769G>A | p.Gly257Arg | Missense | f* |
|  | p16 | Intron 28 | c.1930-2A>C | / | Splice | f* |
|  | p17 | Exon 51 | c.4369G>A | p.Asp1457Asn | Missense | f*# |
|  | p18 | Exon 11 | c.769G>A | p.Gly257Arg | Missense | f* |
|  | p19 | Exon 19 | c.1243C>T | p.Arg415* | Nonsense | f* |
|  | p20 | Exon 31 | c.2089C>T | p.Arg697* | Nonsense | f* |
|  | p21 | Exon 6 | c.484C>T | p.Gln162* | Nonsense | m* |
|  | p22 | Exon 26 | c.1787G>C | p.Gly596Ala | Missense | m*# |
|  | p23 | Exon 7 | c.579delT | p.Gly194Valfs*71 | Frameshift | m* |
|  | p24 | Exon 48 | c.3655G>A | p.Asp1219Asn | Missense | f* |
|  | p25 | Exon 27 | c.1865delC | p.Pro622Leufs*144 | Frameshift | f* |
|  | p26 | Exon 2 | c.157-158delTG | p.Trp53Glufs*19 | Frameshift | m* |
|  | p27 | Exon 2 | c.268G>T | p.Glu90* | Nonsense | m* |
|  | p28 | Exon 17 | c.1072C>G | p.Gln358Glu | Missense | m*# |
|  | p29 | Exon 13 | c.898C>T | p.Gln300* | Nonsense | f* |
|  | p30 | Exon 7 | c.573_574delinsG | p.Pro193Leufs*72 | Frameshift | f* |
|  | p31 | Exon 48 | c.3559G>T | p.Gly1187Cys | Missense | m* |
|  | p32 | Intron 19 | c.1299+1G>A | / | Splice | f* |
|  | p33 | Exon 21 | c.1375C>T | p.Pro459Ser | Missense | m* |
|  | p34 | Exon 2 | c.144delT | p.His48Glnfs*26 | Frameshift | m* |
|  | p35 | Exon 45 | c.3328delC | p.His1110Thrfs*129 | Frameshift | m* |
|  | p36 | Exon 51 | c.4363G>A | p.Gly1455Ser | Missense | m*# |
|  | p37 | Exon 17 | c.1148G>C | p.Gly383Ala | Missense | f*# |
|  | p38 | Intron 21 | c.1461+2dupT | / | Splice | m* |
|  | p39 | Intron 29 | c.1984-2A>G | / | Splice | f* |
|  | p40 | Exon 9 | c.658C>T | p.Arg220* | Nonsense | f*# |
|  | p41 | Intron 12 | c.858+2T>A | / | Splice | f* |
|  | p42 | Exon 17 | c.1085G>C | p.Gly362Ala | Missense | f*# |
|  | p43 | Exon 48 | c.3540delC | p.Gly1181Alafs*58 | Frameshift | m* |
|  | p44 | Exon 35 | c.2434G>A | p.Gly812Ser | Missense | m* |
|  | p45 | Exon 11 | c.757C>T | p.Arg253* | Nonsense | f* |
|  | p46 | Exon 49 | c.3824G>A | p.Trp1275* | Nonsense | f* |
|  | p47 | Exon 46 | c.3421C>T | p.Arg1141* | Nonsense | m* |
|  | p48 | Exon 2 | c.268G>T | p.Glu90* | Nonsense | m* |
|  | p49 | Exon 15 | c.977G>A | p.Gly326Asp | Missense | m* |
|  | p50 | Intron 9 | c.697-2_697-1delAG | / | Splice | f* |
|  | p51 | Exon 41 | c.3008delC | p.Pro1003Leufs*105 | Frameshift | f* |
|  | p52 | Exon 42 | c.3076C>T | p.Arg1026* | Nonsense | m* |
|  | p53 | Exon 1 | c.91C>T | p.Gln31* | Nonsense | f* |
|  | p54 | Exon 41 | c.2991delT | p.Gly998Valfs*110 | Frameshift | f* |
|  | p55 | Exon 23 | c.1522G>A | p.Ala508Thr | Missense | f*# |
|  | p56 | Exon 33 | c.2299G>A | p.Gly767Ser | Missense | f*# |
|  | p57 | Exon 7 | c.579delT | p.Gly194ValfsX71 | Frameshift | f* |
| *COL1A2* | p58 | Exon 40 | c.2450G>T | p.Gly817Val | Missense | m*# |
|  | p59 | Exon 17 | c.838G>A | p.Gly280Ser | Missense | f*# |
|  | p60 | Exon 33 | c.1981G>T | p.Gly661Cys | Missense | f* |
|  | p61 | Intron 26 | c.1557+3A>G | / | Splice | m* |
|  | p62 | Exon 49 | c.3313G>A | p.Gly1105Ser | Missense | f* |
|  | p63 | Exon 31 | c.1801G>A | p.Gly601Ser | Missense | m* |
|  | p64 | Exon 40 | c.2441G>A | p.Gly814Glu | Missense | m* |
|  | p65 | Exon 19 | c.1009G>A | p.Gly337Ser | Missense | m*# |
|  | p66 | Exon 4 | c.110A>G | p.Asp37Gly | Missense | m* |
|  | p67 | Exon 46 | c.3034G>A | p.Gly1012Ser | Missense | f* |
|  | p68 | Exon 35 | c.2081G>A | p.Gly694Asp | Missense | m*# |
|  | p69 | Exon 19 | c.946G>A | p.Gly316Ser | Missense | f*# |
|  | p70 | Exon 52 | c.4065_4066delAT | p.Glu1355Aspfs*19 | Frameshift | f* |
|  | p71 | Exon 29 | c.1666G>T | p.Gly556Cys | Missense | f* |
|  | p72 | Exon 19 | c.1009G>A | p.Gly337Ser | Missense | m* |
|  | p73 | Exon 17 | c.812G>A | p.Gly271Asp | Missense | f*# |
|  | p74 | Exon 28 | c.1648G>A | p.Gly550Ser | Missense | f* |
|  | p75 | Exon 19 | c.1009G>A | p.Gly337Ser | Missense | f*# |
|  | p76 | Exon 17 | c.847G>A | p.Gly283Ser | Missense | f* |
|  | p77 | Exon 19 | c.1009G>A | p.Gly337Ser | Missense | m* |
|  | p78 | Exon 17 | c.847G>A | p.Gly283Ser | Missense | m* |
|  | p79 | Exon 19 | c.964G>A | p.Gly322Ser | Missense | f* |
|  | p80 | Exon 43 | c.2827G>A | p.Gly943Arg | Missense | f*# |
|  | p81 | Exon 26 | c.1504G>A | p.Gly502Ser | Missense | m* |
|  | p82 | Exon 38 | c.2314G>A | p.Gly772Ser | Missense | f*# |
|  | p83 | Exon 50 | c.3583T>C | p.Cys1195Arg | Missense | m* |
|  | p84 | Exon 9 | c.388G>T | p.Gly130Cys | Missense | f* |

p, proband; f, father; m, mother; *, performed gene sequencing; #, nonpenetrance.
