# Supplementary figures and images for: Comparing Clinical and Genetic Characteristics of De Novo and Inherited COL1A1/COL1A2 Variants in a Large Chinese Cohort of Osteogenesis Imperfecta
Source: Front Endocrinol (Lausanne). 2022 Jul 14;13:935905. doi: 10.3389/fendo.2022.935905 (PMC9329653; doi:10.3389/fendo.2022.935905)

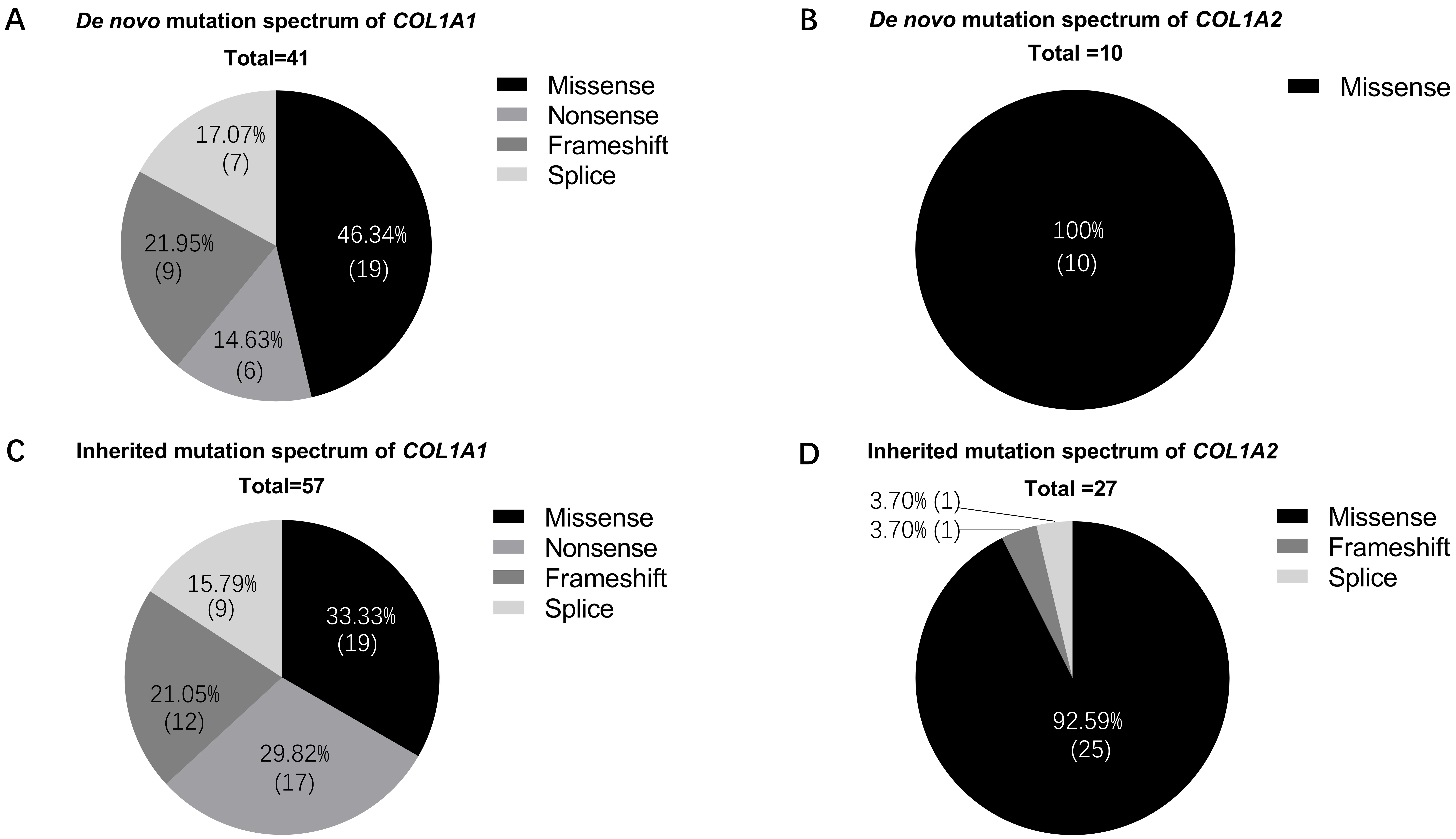

Supplement: Supplementary Figure 1 — De novo and inherited mutation spectrum of COL1A1/COL1A2. (A) De novo mutation spectrum of COL1A1. (B) De novo mutation spectrum of COL1A2. (C) Inherited mutation spectrum of COL1A1. (D) Inherited mutation spectrum of COL1A2. [file DataSheet_1.zip › Supplementary material/Supplementary Figure1.tif]
